# Causal genes identification of giant cell arteritis in CD4+ Memory t cells: an integration of multi-omics and expression quantitative trait locus analysis

**DOI:** 10.1007/s00011-024-01965-7

**Published:** 2025-01-07

**Authors:** Qiyi Yu, Yifan Wu, Xianda Ma, Yidong Zhang

**Affiliations:** 1 Carnegie Mellon University, Pittsburgh, USA; 2 Mudi Meng Honors College, China Pharmaceutical University, Nanjing, China; 3 Queen’s Belfast University, Belfast, Northern Ireland UK

**Keywords:** Giant cell arteritis, CD4+ Memory T cells, Single-cell, Mendelian randomization, Multi-omics

## Abstract

**Background:**

Giant cell arteritis (GCA) is a prevalent artery and is strongly correlated with age. The role of CD4+ Memory T cells in giant cell arteritis has not been elucidated.

**Method:**

Through single-cell analysis, we focused on the CD4+ Memory T cells in giant cell arteritis. eQTL analysis and mendelian randomization analysis identified the significant genes which have a causal effect on giant cell arteritis risk. CD4+ Memory T cells were subsequently divided into gene-positive and gene-negative groups, then further single-cell analysis was conducted. Mendelian randomization of plasma proteins, blood-urine biomarkers and metabolites were also performed. Eventually, the PMA induced Jurkat cell lines were used for biological experiments to explore the specific functions of significant causal genes in CD4+ Memory T cells.

**Results:**

Similarity of CD4+ Memory T cells in GCA and old samples were explored. DDIT4 and ARHGAP15 were identified as significant risk genes via mendelian randomization. The CD4+ Memory T cells were then divided into DDIT4 ± or ARHGAP15 ± groups, and further single-cell analysis indicated the differences in aspects involving intercellular communication, functional pathways, protein activity, metabolism and drug sensitivity between positive and negative groups. In vitro experiments, including overexpression and knockdown, demonstrated that DDIT4 leading to a chronic, low-intensity inflammatory state in CD4+ Memory T cells, eventually promoting the development of GCA.

**Conclusion:**

DDIT4 and ARHGAP15 have significant causal effects on giant cell arteritis risk. Specifically, DDIT4 exhibit pro-inflammatory effects on GCA via promotes chronic, low-intensity inflammatory in CD4+ Memory T cell.

**Supplementary Information:**

The online version contains supplementary material available at 10.1007/s00011-024-01965-7.

## Introduction

Giant cell arteritis (GCA) is a prevalent form of arteritis that mainly poses threats to medium and large arteries. GCA primarily affects older adults, as the highest incidence of GCA is observed in individuals aged 50–80 [1, 2]. Complications, high probability of recurrence, and delays in medical attention further complicate treatment and worsen survival outcomes [3, 4]. GCA presents a wide range of clinical symptoms, including severe headaches, vision disturbances, jaw pain, and limb claudication. These symptoms can occur early in the disease course, and make diagnosis challenging [5, 6]. Currently, the temporal artery biopsy is considered as the “gold standard” [7]. The complexity of GCA symptoms and its diagnostic challenges highlight the urgent need for further research.

CD4+ T cells play a key role in regulating the autoimmune response. Through various functional pathways, CD4+ T cells are activated upon recognizing antigens, sustaining and promoting the inflammatory response through the release of proinflammatory cytokines and interactions with various immune cells [8]. Meanwhile, during GCA development, CD4+ T cells were observed to infiltrate protected niches within the vessel wall, differentiate into cytokine-producing cells, establish residency within tissues, and eventually induce macrophages transformation into effector cells that contribute to tissue damage [9]. Therefore, CD4+ T cells are not only one of the significant key cell clusters that trigger inflammatory responses, but also the important regulators of immune cell populations in GCA development. However, the heterogeneity of CD4+ T cells suggested the varied functions of CD4+ T cell subtypes in GCA.

DNA Damage Inducible Transcript 4 (DDIT4), also named REDD1, regulates cell growth, proliferation, and survival by inhibiting the activity of rapamycin complex 1 (mTORC1). During inflammation response, the cells were stimulated, and endoplasmic reticulum stress induces cell apoptosis through mTOR pathway. DDIT4 was reported to be upregulated in stimulated T cells in inflammation response, protecting T cells from the mTORC1-mediated apoptosis [10–12]. Parallelly, in an LPS-induced shock model, DDIT4 knockdown mice exhibited reduced inflammation, which indicated the potential pro-inflammatory function of DDIT4 [13–15]. In GCA, the CD4+ T cells play a significant role in autoimmune response, in which DDIT4 might be a potential contributor in the dysregulation of vascular inflammation.

Rho GTPase activating protein 15 (ARHGAP15) is a negative regulator of Rac1 (Ras-related C3 botulinum toxin substrate 1). Dysregulation of Rho-GTPase leads to the accumulation and death of CD4+ T cells in inflammation, and the downregulation of ARHGAP15 effectively ameliorates inflammatory symptoms, particularly in senescent samples [16, 17]. The association of GCA with aging, along with the reduced inflammation through ARHGAP15 downregulation, suggests that ARHGAP15 may play a key role in modulating inflammation in GCA.

Here, we conducted transcriptome analysis and utilized the mendelian randomization (MR) method to examine and validate the causal association between genes linked to CD4+ Memory T cells and occurrence of GCA. DDIT4 and ARHGAP15 were identified as critical genes. Using Jurkat cell line, the function of DDIT4 was further explored in experiment level. This novel research sheds light on advancing GCA investigations and potentially facilitate the development of more targeted treatments for this intricate autoimmune disease.

## Methods

### Data acquisition

**Fig. 1:**
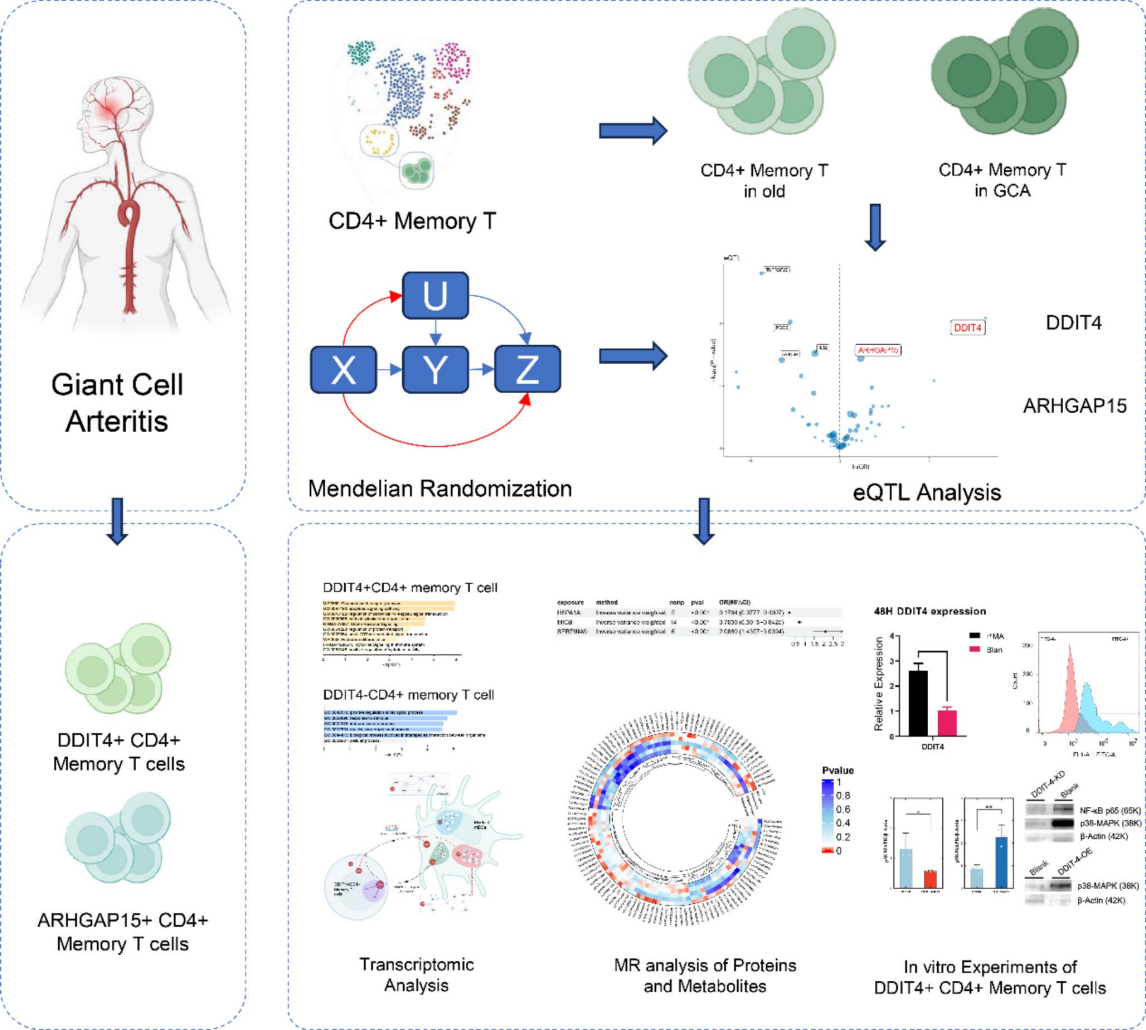
The flowchart of research

Our workflow is illustrated in Fig. 1. Gene Expression Omnibus (GEO) (http://www.ncbi.nlm.nih.gov/geo) was searched for datasets. The single-cell data GSE157007 and GSE198891 were profiled using GPL24676 platform [18, 19]. The bulk-seq data GSE174694 profiled using GPL18573 platform, which contains 40 GCA samples and 31 normal samples, was downloaded for external validation [20]. Expression quantitative trait locis (eQTLs) data were obtained from ieu database (https://gwas.mrcieu.ac.uk/). From the ieu database, we acquired finn-b-M13_GIANTCELL, a GWAS dataset that contains 213,145 controls and 459 cases of giant cell arteritis for MR analysis. Additionally, the validation dataset of the GCA finn-b-GIANT_CELL_TEMP_ARTERITIS was also acquired, which contains 217,965 controls and 421 cases. The protein quantitative trait locis (pQTLs) data was retrieved from deCODE genetics (https://www.decode.com/summarydata/), including plasma protein levels measured with 4907 aptamers in 35,559 Icelanders.

Meanwhile, from the Canadian Longitudinal Study of Aging (CLSA) cohort, the GWAS data containing 1400 blood metabolites was acquired [21]. The cohort measured 1400 human metabolites in plasma samples obtained from 8299 randomly selected, unrelated European ancestry samples.

The exposure data involving 35 blood and urine laboratory measurements were downloaded from the UK biobank, published by Armstrong et al. [22] in 2021 in Nature Genetics. In the GWAS data, 363,228 Europeans were included after rigorous quality control. The study incorporated a total of 2.1 million SNP loci and 35 blood and urine metabolites for a comprehensive genome-wide association analysis. Detailed information on the datasets was presented in Table 1.

**Table 1:** The detailed information of adopted datasets

Dataset	Categorize	Uploader/Website	Samples
GSE198891	Single-cell seq	Reitsema et al	GCA
			GCA(n = 3)
			Healthy (n = 3)
GSE157007	Single-cell seq	Luo et al	Aging
			Frail (n = 5)
			Old (n = 6)
			Healthy (n = 3)
GSE174694	Bulk-seq	Buback et al	GCA
			GCA (n = 40)
			Healthy (n = 31)
finn-b-M13_GIANTCELL	eQTL	Ieu database	Control (n = 213,145)
			Case (n = 459)
finn-b-GIANT_CELL_TEMP_ARTERITIS	eQTL	Ieu database	Control (n = 217,965)
			Case (n = 421)
Large-scale integration of the plasma proteome with genetics and disease	pQTL	Ferkingstad et al	n = 35,559
Genetics of 35 blood and urine biomarkers in the UK Biobank	Biomarker GWAS data	Yosuke Tanigawa et al	n = 363,228
CLSA Cohort	Metabolite GWAS data	Chen et al	n = 8299

### Single-cell data process and cell annotation

“Seurat” package (ver 4.3.0) was employed for single-cell data quality control and downstream analysis [23]. To screen out low quality cells, the quality control thresholds were set as 200 < nFeature_RNA < 4000 and percentage.mt < 10%. Normalization process was conducted via “LogNormalize” method with a scale factor of 10,000, after which the top 2000 varied genes were identified. After expression level scaling, we implied the principal component analysis (PCA) and then the “Harmony” (ver 0.1.1) package was introduced to perform the batch effect correction and integration [24]. The Uniform Manifold Approximation and Projection (UMAP) method was used for dimension reduction and cell clustering.

### Cell annotation and extraction of T cell clusters

Next, we annotated cell groups according to canonical marker genes as shown in Table 2. Gene expression of each cluster was visualized using dot plots. T cells were extracted from the annotated scRNA data, and then the dimension reduction and unsupervised clustering were implemented again. The subtypes of T cells were annotated according to previous publications. The marker genes were presented in Table 3. After extracting T cells, the characteristic genes in different sub-clusters were identified using “FindMarkers” function, and the on which basis the functional enrichment was performed via “scRNAtoolVis” package (ver 0.0.6, https://github.com/junjunlab/scRNAtoolVis). To identify the pathologic cell types of GCA, we applied the scPagwas algorithm (ver 1.3.1, https://github.com/sulab-wmu/scPagwas) to integrate GWAS data finn-b-M13_GIANTCELL from ieu database with annotated single-cell dataset. A trait-relevant score (TRS) of each cell is computed by averaging the expression level of the trait-relevant genes and subtracting the random control cell score via the cell-scoring method used in Seurat. Meanwhile, to identify the “trigger cell” of GCA, we employed “Augur” package (ver 1.0.3, https://github.com/neurorestore/Augur) [25]. Augur is an R package to prioritize cell types involved in the response to a specific perturbation within high-dimensional single-cell data. Such prioritization can illuminate the contribution of each cell type to the disease state, which provide guidance to downstream research.

**Table 2:** The canonical marker genes for cell type distinction and annotation in scRNA samples

Cell Type	Marker
Myeloid mDCs	LGALS1, IL1B, LYZ, S100A9
T cells	CD3D, CD8A, CD8B, IL7R, CCR7
NK cells	NKG7, GZMB, GNLY, KLRB1
B cells	CD79A, CD74, MS4A1
NC Monocytes	FCGR3A, LGALS1
Platelets	PPBP
pDCs	CD74, GZMB, TCF4

**Table 3:** The detailed canonical marker genes for T cell type distinction and annotation

Cell Type	Marker
Naive T	CCR7, TCF7, SELL, LTB, HLA-B
CD4+ Naive T	CD4, CCR7, TCF7, SELL, LTB, HLA-B
CD4+ Memory T	CD44, LTB, IL32, CCR6
CD8 + Naive T	CD8A, CD8B, SELL, LTB, CCR7, TCF7
CD8 + Effector T	CD8A, FASLG, FAS

### Jaccard similarity analysis

In GCA and old samples, the composition of T cells were similar. Thus, the signature genes of two cell types were used to compare the transcriptional similarity and calculate the Jaccard similarity coefficient [26]. We assessed transcriptional similarities between different T subclusters by calculating the Jaccard similarity coefficient using the characteristic genes. Selecting the most stable proportion of T subclusters in GCA and old samples, CD8 + Naive T cells, as the reference, the functional enrichment analysis was conducted based on the upregulated characteristic genes identified in CD4+ Memory T cells-reference group, to evaluate the functional similarity of CD4+ Memory T cells between GCA patients and the old.

### Pseudotime inference and communication analysis of T cells

“Slingshot” package (ver 2.6.0) was used to infer T cells state trajectories [27]. Slingshot is a novel tool for inference of multiple branching lineages. It consists of two main steps: global lineage structure inference based on minimum spanning tree (MST) construction, and inference of pseudotime variable for cells in each lineage by fitting smooth branching curves to these lineages. The two-step structure endows Slingshot with high robustness to noisy single-cell datasets, allowing it to infer trajectories and stages of cell differentiation in multiple branching lineages from complex single-cell data. Meanwhile, the “Monocle” (ver 2.26.0) was also utilized for the pseudo-time analysis. 2000 random T cells were extracted from GCA and old samples and selected with criteria of mean expressions > 0.1 and dispersion empirical > 1*dispersion fit cells. After dimensional reduction, the cell differentiation tracks were visualized [28].

We employed the “Cellchat” (ver 1.6.1) package to analyze cell-state specific signaling communications between annotated cell clusters in samples [29]. Specific intercellular interactions between each cell cluster were identified with prior knowledge of the interactions between signaling ligands, receptors and their cofactors. The probability of cell–cell interactions was inferred subsequently. After random permutation of cell labels, the probabilities were recalculated and visualized through a weighted directed graph representing the intercellular communication network.

### eQTL MR analysis

Using “FindMarkers” function, we identified differentially expressed genes (DEGs) in CD4+ Memory T cells, which were used as exposures in the MR analysis. In brief, the DEGs between CD4+ Memory T cells and other T subclusters were first identified, then the DEGs between CD4+ Memory T cells and all other cells were identified. The input DEGs were the intersection of CD4+ Memory T cells-other T subcluster DEGs and CD4+ Memory T cells-all other cell cluster DEGs. The functional enrichment of the input DEGs was conducted using Metascape (https://metascape.org/). After identifying the input eQTL instrument variables, “TwosampleMR” package (version 0.5.7) [30] was employed to perform the MR analysis on GCA data finn-b-M13_GIANTCELL and finn-b-GIANT_CELL_TEMP_ARTERITIS from ieu database. The criteria for SNP quality control were set as follows: (1) European ancestry to reduce potential bias from population stratification. (2) The R^2^ [R^2^ = 2*EAF*(1-EAF)*Beta^2^, EAF: Effect Allele Frequency; Beta: Effect size] of each SNP was calculated summed them up to estimate the overall F statistics [F = R^2^*(N − 2)/(1 − R^2^), N: number of samples in the exposure GWAS data]. Instrumental variants with low F-statistics were considered weak instruments and deleted. (3) To remove genetic linkage between SNPs, a reference panel from the 1000 Genomes Project was adopted [31, 32]. The thresholds were set as follows: linkage disequilibrium (LD) r^2^ = 0.001 and cropping range of 10,000 kb. Eventually, after quality control of instrumental variants, the significant SNPs (*p* < 5 × 10^–8^) that were associated with input DEGs were selected. The processed SNPs were then integrated and harmonized with outcome data using inbuilt function “harmonise_data”.

In the main MR analysis, the differentially expressed genes in CD4+ Memory T cells were considered as exposures, and GCA GWAS data was treated as the outcome. Using “TwoSampleMR” package, the MR analysis was performed and odds ratio with 95% confidence intervals of each exposure to GCA outcomes was calculated. Respectively, 5 methods, including MR-Egger, weighted median, inverse variance weighted (IVW), simple mode, and weighted mode, were applied to estimate the MR effects of each exposure. The Wald ratio was adopted for genes with only one available instrument variant. IVW is based on the assumption that all selected instruments are valid, which possesses the highest statistical power of among all methods [32]. Given that pleiotropy between exposures and outcomes may introduce bias, a horizontal pleiotropy test was conducted on the eQTL and GWAS data. Additionally, the robustness of the study and potential heterogeneity were investigated via the Cochran’s Q test [33]. The exposure genes were further validated in finn-b-GIANT_CELL_TEMP_ARTERITIS dataset to verify the significant causal effect to GCA risk.

To further explore the direction of causation between selected exposures and outcomes, the reverse-MR analysis was performed. Eventually, Steiger Filtering was employed to compare the SNP-explained outcome variance and SNP-explained exposure variance, and genes with more outcome variance were excluded.

### pQTL and metabolite MR analysis

Apart from eQTL, we also conducted MR analysis on protein and metabolite level. The plasma proteome GWAS data were acquired from deCODE genetics (https://www.decode.com/summarydata/), the instrumental variants from GWAS data were extracted at criterion of *p* = 5 × 10^–8^. The two-sample MR analysis was conducted to estimate the associations between plasma protein levels and GCA risk. For association estimates of each plasma protein, the Bonferroni adjustment was performed. Subsequently, functional enrichment was performed on proteins that were identified to be causal to GCA occurrence using proteomaps (https://proteomaps.net/).

To infer the activities of different proteins in specific CD4+ Memory T cell clusters, “metaVIPER” (https://github.com/califano-lab/single-cell-pipeline) was used [34]. metaVIPER is a statistical framework that was developed to estimate protein activity from gene expression data using enriched regulon analysis performed by the algorithm aREA [35]. The regulatory network of proteins and their transcriptional targets, as well as the likelihood of their interactions was inferred f by the algorithm ARACNe [36]. metaVIPER uses multiple gene regulatory networks to infer protein activity, and each individual gene regulatory network are finally integrated to a consensus activity estimation.

The MR analysis was also performed to estimate the associations between genetically predicted metabolite levels and GCA risk as well as 35 blood-urine biomarkers and GCA risk. The instrumental variants were extracted from CLSA cohort (metabolomes) and UK Biobank (blood and urine biomarkers) at p = 5 × 10^–8^, and the Wald ratio (for single instrument variant) or a combination of 5 methods (MR-Egger, IVW, weighted median method, weighted mode and simple mode for multiple instrument variants) were adopted for main MR analysis.

### Colocalization analysis and phenome‑wide association analysis

An SNP may occasionally be located within multiple gene regions. If an SNP contained eQTL information for more than one gene, the causal effect on the disease might be driven by different genes, and the colocalization analysis was further adopted to identify the shared or distinct genetic variants of GCA and eQTLs. Here, for a given SNP, the probability that the SNP is linked to GCA is assigned as p_1_, the probability that the SNP is linked to a significant eQTL is p_2_, and the probability that the SNP is both linked to GCA and eQTL is denoted as p_12_. The “coloc” (ver 5.1.0) package provides default priori probabilities: p_1_ = 1 × 10^–4^, p_2_ = 1 × 10^–4^ and p_12_ = 1 × 10^–5^ [37]. Meanwhile, the package derives posterior probabilities for five hypotheses (H0, H1, H2, H3, H4) after colocalization analysis to identify whether an SNP is shared between exposure and outcome. The five hypotheses: PP_H0_, the SNP is neither associated with gene expression nor GCA risk; PP_H1_: the SNPs are associated with gene expression, while not GCA risk; PP_H2_: the SNP is associated with GCA risk, while not gene expression; PP_H3_: the SNP is associated with both gene expression and GCA risk, driven by different causal variants; PP_H4_: the SNP is associated with both gene expression and GCA risk, driven by a shared causal variant.

Meanwhile, to explore the association between selected exposure SNPs of identified significant causal genes and existing phenotypes, we also performed the phenome-wide association study (PheWAS) via PhenoScanner GWAS Database (http://phenoscanner.medschl.cam.ac.uk) [37, 38]. The PhenoScanner database provides public results from over 65 billion associations and over 150 million unique genetic variants. Through PheWAS, we investigated the associations between disease phenotypes and selected SNPs with genome-wide significance (*p* < 5 × 10^–8^) [39].

### Significant genes identified in CD4+ memory T cells

After the MR analysis, we visualized the expression level of selected genes in different cell types, and the expression distribution of significant risk genes in the single-cell sequence data, visualized through “schex” (https://github.com/SaskiaFreytag/schex) and “Scillus” (https://github.com/xmc811/Scillus) packages. We also adopted the “GeneSwitches” (https://github.com/SGDDNB/GeneSwitches) method to investigate the on/off state of genes in CD4+ T cells [40]. GeneSwitches binarizes the gene expression as either an “on” or “off” state and identifies the order in which functional ontologies are acquired or lost during a transition. Meanwhile, we utilized “monocle” package to explore the variation in expression levels of identified significant causal genes in T cell differentiation of GCA samples.

According to the expression or non-expression of significant genes, the CD4+ Memory T cells were divided into gene-positive and gene-negative groups. Additionally, the DEGs between gene-positive group and gene-negative group were identified through “FindMarkers” function, and the functional enrichment was performed via Metascape.

Furthermore, to identify the potential transcriptomic factor (TF) regulatory networks in CD4+ Memory T cell subtypes, we performed SCENIC (ver 1.3.1) analysis [41]. The GENIE3 was used for identification of TF targets via constructing co-expression network, then the enrichment of TF-motif and discovery of direct TF target were completed by RcisTarget. The reference genome dataset was acquired from cisTarget Database (https://resources.aertslab.org/cistarget/). Eventually, the score of regulon activity in each cell was calculated to estimate the intensity of different TFs in CD4+ Memory T cell subtypes.

Comparing to Cellchat, “CytoTalk” (https://github.com/tanlabcode/CytoTalk) provides a novel method to investigate the specific ligand-receptor communication in GCA cell clusters [42]. CytoTalk first constructs an integrated gene network as a topological skeleton, including intra- and intercellular gene interactions. In the network, node weights are defined as rewards (cell-specific gene activity) and edge weights are defined as costs (interaction probabilities between two genes). The signaling network between the two cell types is identified by solving the prize-collecting Steiner Forest (PCSF) problem in this empowered integrated network. The goal of the PCSF problem is to find an optimal sub-network in the integrated network that contains genes with a high level of cell-type specific expression and a strong association with highly active ligand-receptor pairs.

The “tricycle” package (ver 1.8.0) was adopted to evaluate the cycle state of each cell in single-cell samples [43]. By assigning scores to each cell based on the gene expression of G/M and S-phase markers, tricycle categorized cells into G1, G2M and S phases based on their cell cycle scores.

#### The metabolism analysis of CD4+ memory T cell subtypes

The “scMetabolism” (ver 0.2.1) package was adopted to evaluate the metabolic diversity of different cell clusters in samples [44]. After scoring each type of cell, the activity scores in various metabolic pathways were obtained based on the conventional single‐cell matrix file.

Specifically, to explore the difference of metabolic flux between the CD4+ Memory T cell subtypes, we performed single cell flux estimation analysis (scFEA, ver 1.1.2) [45]. Through generating a graph neural network based on inputted single-cell matrix, module gene file and the stoichiometry matrix of relationship between compounds and modules, scFEA characterizes the flux intensity of metabolites in different CD4+ Memory T subgroups.

#### Drug response of different T subclusters

Due to the heterogeneity among cell populations, cellular responses to drugs vary from one to another. We used “scRANK” package (https://github.com/ZJUFanLab/scRank) to predict the drug response rank of different T subclusters in GCA [46]. scRANK employs a target-perturbed gene regulatory network to rank drug-responsive cell populations via calculating the perturbations introduced by drugs in the network using single-cell transcriptomic data.

#### Bulk-seq validation and friend analysis

Bulk-seq data GSE174694, which contains 40 GCA samples and 31 normal samples, was downloaded from GEO for external validation. The normalization was implemented using “limma” (ver 3.54.0) package, and the expression levels of significant genes in GCA group and normal group were visualized through “ggpubr” (ver 0.6.0) package (https://rpkgs.datanovia.com/ggpubr/index.html). Moreover, we investigated the diagnostic ability of significant genes and the typical markers of GCA via Receiver Operating Characteristic (ROC) curves [47]. Also, the friend analysis was conducted. Semantic similarity of GO terms enriched by significant genes was calculated using the Wang method via “GOSemSim” package (ver 2.22.0) [48].

#### Cell transduction and incubation

The Jurkat T cell lines were obtained from Cellcook Bio. They were cultured in culture medium (RPMI 1640) with 10% Fetal bovine growth serum (FBS) and penicillin–streptomycin-amphotericin B (Penicillin–Streptomycin–Amphotericin B Solution, 100X, Beyotime, C0224), 37 °C and humidified atmosphere containing 5% CO_2_. To perform the overexpression and knockdown of DDIT4, the pIRES2-EGFP-DDIT4 and pKDL-DDIT4 plasmids were constructed and then were integrated into Jurkat cells (details in Supplement Material). After transduction, cells were subsequently incubated with or without 5 μM Phorbol-12-Myristate-13-Acetate (PMA, Beyotime, S1819) to activate Jurkat cells.

#### Quantitative real-time polymerase chain reaction (qRT-PCR)

Total RNA was extracted using the RNA isolation kit (Vazyme, R711-2), and reverse transcription was performed using the HiScript III RT SuperMix for qPCR (+ gDNA wiper) kit (Vazyme, R211-01). The specific amplification conditions are set according to the instructions provided by the supplier. The cDNA served as the template to detect mRNA expression of genes. The relative gene expression of OE/KD and blank cells before/after PMA induction was calculated by 2^−ΔΔCt^ method. The specific primers are presented in Table 4. GAPDH was chosen as the internal control (Beyotime, QH00009S).

**Table 4:** The primers used in research

Primer	Sequence or manufacturer
IL-1β	Beyotime, QH03645S
IL-2	Beyotime, QH03645S
IL-6	Beyotime, QH03645S
IL-17A	Beyotime, QH03769S
TNF-α	Beyotime, QH05997S
DDIT4	Beyotime, QH08689S

#### Western blot (WB) assays

To detect the expression of selected genes, the proteins were extracted from Jurkat cells after centrifugation and lysis in RIPA-PMSF (RIPA Lysis Buffer, PMSF Solution (100 mM), Beyotime, P0013C, ST507) buffer on ice. The protein samples were quantified using the BCA method (BCA Protein Quantification Kit, Vazyme, E112), whereby the samples were heated at 95 °C Celsius for 10 min after the addition of the loading buffer (Loading buffer (DTT), HANGZHOU FUDE BIOLOGICAL TECHNOLOGY, FD002). A weight of 40 or 50 µg was sampled per well. Additionally, the supernatant of cell culture medium was also collected for detection of secreted inflammatory factors. The collected proteins were subsequently separated through SDS-PAGE, and transferred onto PVDF membranes (Immobilon® -P PVDF Membrane, Merck, IPVH00010). The membranes were incubated with specific primary antibodies, and followed by incubation of the appropriate peroxidase-conjugated secondary antibodies. Detailed information of the antibodies is provided in Table 5. GAPDH and beta-Actin was chosen as the internal control (GAPDH-Elabscience, E-AB-40337, beta-Actin-Elabscience, E-AB-40338).

**Table 5:** The antibodies used in research

Antibody	Sequence or manufacturer
DDIT4	Huabio, HA722161
MCL1	Huabio, ET1606-14
IL-6	Huabio, R1203-2
IFN-γ	Sanying, 15365-1-AP
GAPDH	Elabscience, E-AB-40337
TNF-α	Huabio, R1203-1
CD45	ab27287

#### Cell counting Kit-8 (CCK-8) assay

To determine the regulatory effect of DDIT4 expression on Jurkat cells, the CCK-8 assay was conducted. Cells were seeded in 96-well plates and cultured in medium containing 10% FBS. A total of 3000 cells were added to each well, and the experiment was conducted after an 8 h incubation period. The number of living cells was determined by CCK-8 assay kit (Cell Counting Kit-8, Beyotime, Shanghai, China, 10 μL/well), and the absorbance was detected at 450 nm using a microplate reader (ThermoFisher).

#### Flow cytometry

For the investigation of the cell differentiation and apoptosis of Jurkat cells, staining solutions CD45-FITC (ThermoFisher, MHCD0429) and Annexin V-FITC/PI (Yeasen Bio, 40302ES) were adopted and operated according to the protocol of manufacturer. 48 h after transfection, the Jurkat cells were collected into tubes and centrifuged. The supernatant was discarded, and the cells were washed with PBS twice. Staining solutions were added to each sample, and analyzed by flow cytometry immediately after a 10–15 min incubation.

#### LDHB assay

Lactate dehydrogenase (LDH), an enzyme stabilized in the cytoplasm of the cell, normally exists only inside the cell. During cell death process, the plasma membrane ruptures and LDH is rapidly released outside the cell. LDH leakage from cells to the culture medium was determined using a Cytotoxicity Detection Kit (Beyotime, C0016). The cell culture supernatant was collected, and a measurement of 100 μL of the supernatant was incubated with 100 μL of the reaction mixture for 30 min at room temperature, protected from light, and the absorbance was then measured at 490 nm.

#### Detection of cytokines in serum-free cell culture media using WB

The knockdown, overexpressed or PMA-stimulated cells were digested using trypsin and inoculated into 24 empty plates at a density of 40,000 cells per well. The knockdown, overexpressed or PMA-stimulated cells were then cultured using serum-free RPMI 1640 medium for a period of 12 h. The supernatant was collected and centrifuged to remove precipitates, after which the medium was concentrated by ultrafiltration by a factor of 10. This was then added a 4 μL volume of loading buffer (Loading buffer (DTT), HANGZHOU FUDE BIOLOGICAL TECHNOLOGY, FD002). A volume of 20 µL of the sample should be added to each well designated for electrophoretic loading. SDS-PAGE was performed, then, transmembrane transfer and detection of expression using corresponding antibodies.

#### Statistical analysis

Bioinformatic analyses were performed using R software, ver 4.3.0 (http://www.rproject.org), Wilcox.test was applied in bioinformatic data analysis. Two-tailed unpaired Student’s t-test was used to compare differences between experimental data. Data were presented as the means ± standard deviation. Statistical significance was defined as a p less than 0.05. *, *p* < 0.05; **, *p* < 0.01; ***, *p* < 0.001; ns, no significance.

## Results

### Cell clusters identification and annotation

The scRNA-seq datasets GSE157007 and GSE198891 were merged into one profile. After quality control, we acquired 74,143 cells for downstream analysis (Fig. S1A–C). Then the normalization and PCA were constructed, and 10 PCs were selected. The “Harmony” package was used to perform the batch effect correction. 21 cell clusters were identified and annotated based on the expression of marker genes (Fig. S1D).

**Fig. 2:**
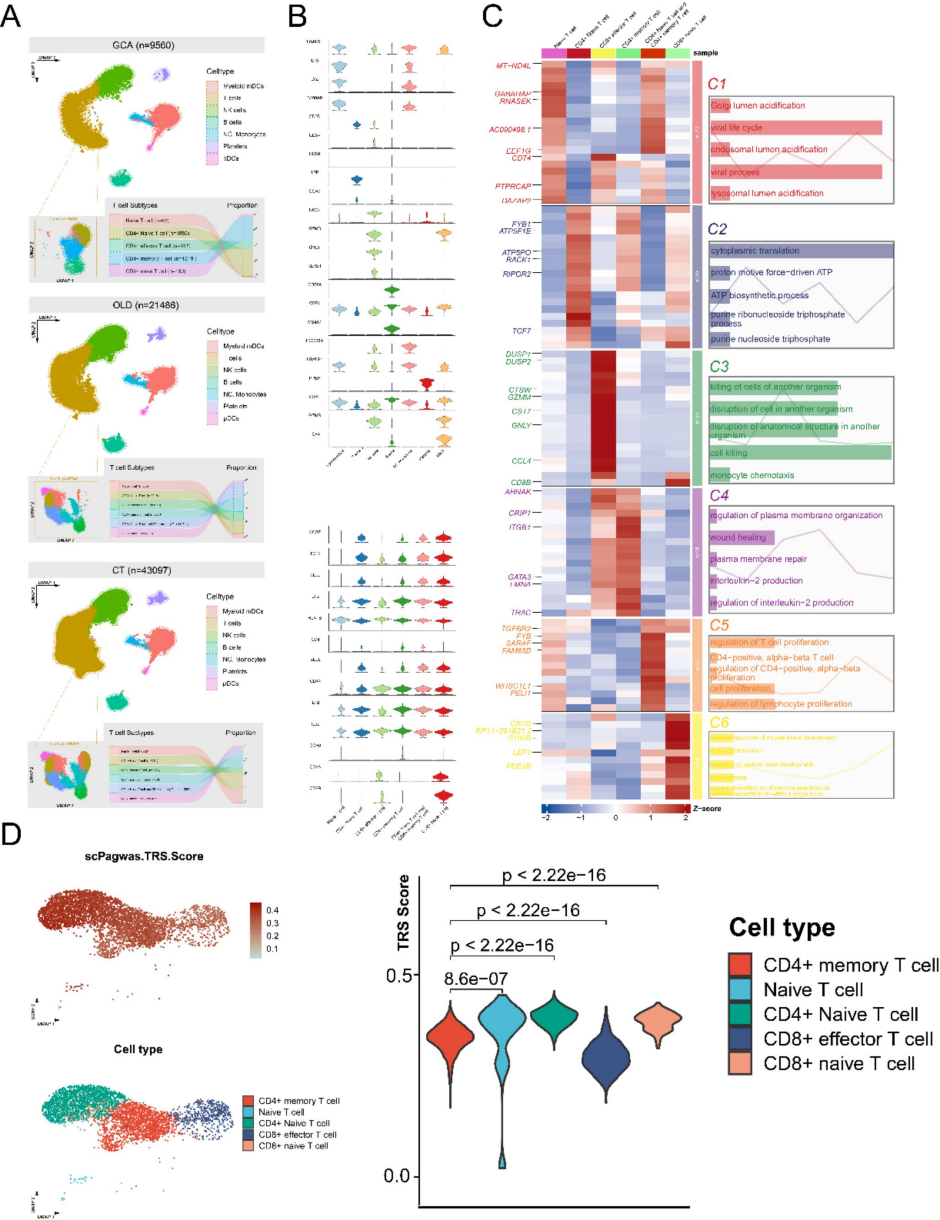
Clusters and proportions of cell from GCA patients, old donors and healthy controls. **A** 21 cell clusters were obtained after PCA analysis, and 7 types of cells were identified by manual annotation. T cells in samples were extracted and re-clustered, 6 types of T cells were subsequently annotated. **B** The average expression level of canonical marker genes of each T cell cluster. **C** Top DEGs in each subclusters and corresponding functional enrichment, n represents the number of top DEGs selected. **D** ScPagwas TRS score of different T cell clusters. CD4+ Naive T cells showed the highest trait relevance with GCA

Main cell types, including Myeloid mDCs (myeloid dendritic cells), T cells, NK cells, B cells, NC Monocytes (non-classical monocytes), Platelets, and pDCs (plasmacytoid dendritic cells) were identified (Fig. 2A). We specifically extracted and re-clustered T cell groups from GCA samples into 13 individual groups (Fig. S1E, F). Detailed annotations based on marker gene expression from previous research identified Naive T cells, CD4+ Naive T cells, CD8 + Naive T cells, CD4+ Memory T cells, and CD8 + Effector T cells (Fig. 2A). Compared to healthy controls, CD4+ Memory T cells showed a significant increase in both the old donor and GCA groups, with the increase being particularly pronounced in the GCA group. The variation in the proportions of cell clusters among healthy control, old donors and GCA patients was visualized using a bar plot. The average expression of canonical marker genes for cell type annotation was visualized in Fig. 2B. After identifying the top upregulated characteristic genes in each T subclusters, the GO functional enrichment was conducted. Pathways related to CD4+ Memory T cells, in C4 group, were mainly enriched in T cell proliferation and interleukin-2 regulation, suggesting the specific alternations of cell activity in GCA (Fig. 2C).

To explore the pathologic cells associated with GCA, we sought to identify specific cell subpopulations that are relevant to GCAs by integrating GWAS summary statistics with single-cell seq data using the scPagwas method (Fig. 2D). Among the main cell types, we found that CD4+ Naive T cells showed relative higher TRSs, compared with other cell types. On contrary, the Augur scores of different T subclusters in old and GCA samples revealed that CD4+ Memory T cells were most insensitive to perturbation of GCA (Fig. S2A). The stable status of CD4+ Memory T cells might indicate their similar role in the transformation progress to both GCA and aging.

### Similarity of CD4+ memory T cells in GCA and old samples

**Fig. 3:**
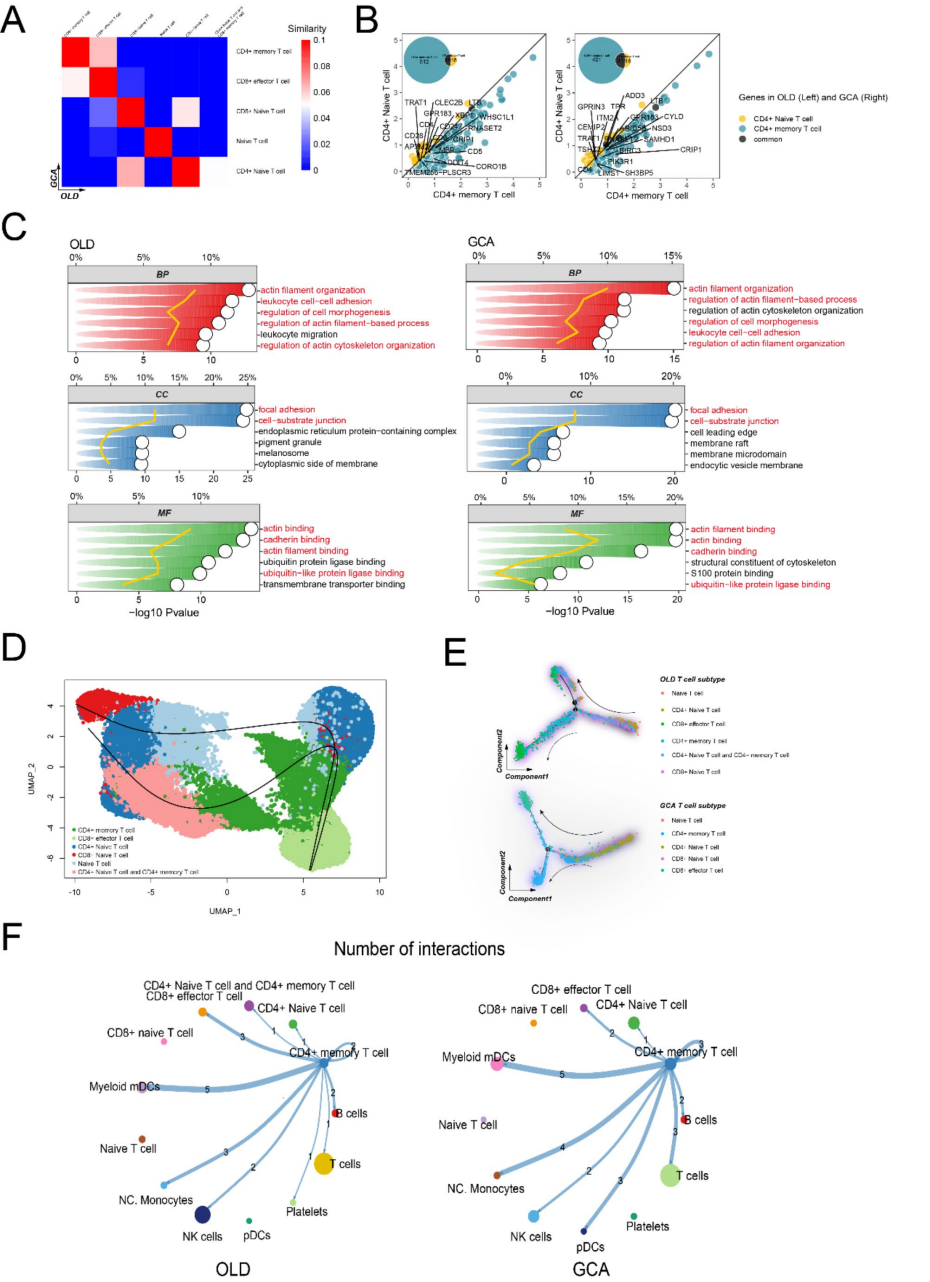
Common characteristics of CD4+ Memory T cells in GCA and old samples. **A** The Jaccard analysis of different T subclusters. **B** Intersection of DEGs of CD4+ Memory T cells-reference and CD4+ Naive T cells-reference, conducted in old samples and GCA samples. **C** The major enriched pathways of intersected genes of DEGs of CD4+ Memory T cells-reference and CD4+ Naive T cells-reference in old samples and GCA samples. **D** The differentiation track of T cell subclusters, analyzed by Slingshot and **E** Monocle. The CD8 + Naive T cells is mainly in the initial differentiation level, as well as the CD8 + effector T cells is mainly in the terminal differentiation level. **F** Intercellular communication of CD4+ Memory T cells in old samples and GCA samples. The colors indicate the sender of cell–cell communications, CD4+ T cells, and the edge thickness represents communication weight, and the arrow marked the communication direction

Given that GCA is more prevalent in the elderly, we speculate that there are potential associations between CD4+ Memory T cells in GCA and the old. We employed “Jaccard” to evaluate the similarities of different T cell subclusters between GCA and the old samples (Fig. 3A). In GCA samples, CD4+ Memory T cells showed high similarity to them in the old samples. The commonly upregulated DEGs were identified using “FindMarkers”. 530 upregulated DEGs were identified between CD4+ Memory T cells and CD8 + Naive T cells in old group, whereas 432 upregulated DEGs were identified between CD4+ Memory T cells and CD8 + Naive T cells in the GCA group (Fig. 3B). An obvious increase of DEGs was observed in both GCA and old groups, which might indicate gene expression variation of CD4+ Memory T cells was quite similar in GCA patients and elderly. Functional enrichment based on the DEGs identified in CD4+ Memory T cells-CD8 + Naive T cells group in GCA and old samples revealed the pathways activated in CD4+ Memory T in two groups (Fig. 3C). In old and GCA groups, similar pathways were enriched, including actin filament regulation, cell–cell adhesion, focal adhesion and actin binding. Thus, it can be assumed that the CD4+ Memory T cells exert similar biological and clinical functions in GCA and old groups.

Furthermore, the similarity of CD4+ Memory T cells in GCA and old samples were explored by cell differentiation and communication analysis. Extracting all T cells in single-cell dataset samples, the cell state trajectories were investigated by “Slingshot” and two different cell trajectories of T cells were revealed (Fig. 3D). The analysis indicated that CD8 + Naive T cells were at the beginning of the trajectories and differentiated into CD8 + Effector T cells in the terminal state. Additionally, the CD4+ Naive T cells shifted towards CD4+ Memory T cells in both trajectories. Extracting T cells in GCA and the old groups, the specific cell differentiation trajectories were identified using “Monocle” method subsequently. The trajectory predicted in GCA and old samples both indicated that CD4+ Memory T cells were in the later-middle stage of differentiation, while CD4+ Memory T cells in GCA showed an individual terminal stage in differentiation trajectory. CD4+ Memory T cells in old samples didn’t show such differentiation trajectory (Fig. 3E).

2000 cells were extracted from the samples for cell–cell communication analysis. Using the “Cellchat” package, we calculated and presented major intercellular communications between various T cell clusters and other cell clusters in GCA and elderly samples (Fig. 3F, S2B). The intercellular communication analysis revealed communication characteristics of CD4+ Memory T cells in GCA and elderly samples. CD4+ Memory T cells showed strong communication activities to myeloid mDCs, and activities in IL16-CD4 and MIF (CD74-CCR4) pathways in both GCA and elderly samples, revealing the similar communication patterns in the GCA and aging process.

### Causal gene identification of GCA risk by MR analysis

**Fig. 4:**
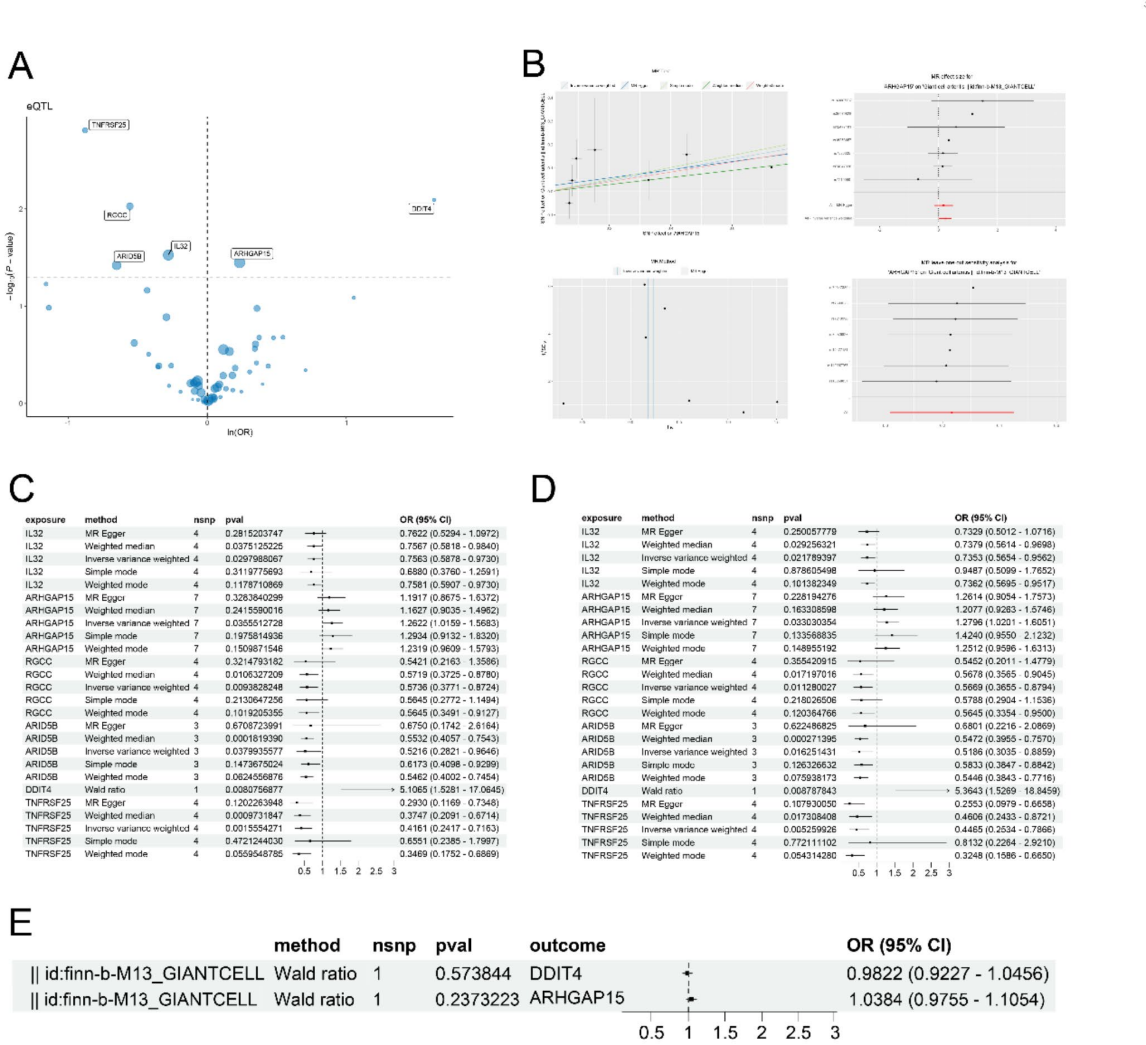
The eQTL MR analysis. **A** The volcano plot presented the genetic effects of differentially expressed genes in CD4+ Memory T cells on GCA, from which 6 genes with significant effects were selected as significant exposures. **B** The MR analysis process of ARHGAP15. Scatter plot showing the causal effect of ARHGAP15 on the risk of GCA; Forest plot showing the causal effect of each SNP on the risk of GCA; Funnel plots visualized overall heterogeneity of MR estimates for the effect of ARHGAP15 on GCA; Leave-one-out plot indicated causal effect of ARHGAP15 on GCA risk when leaving one SNP out. **C** The MR estimates of significant exposures on GCA in test dataset. **D** The MR estimates of significant exposures on GCA in validation dataset. **E** Reverse mendelian analysis on causal effect between GCA and DDIT4/ARHGAP15, which set GCA as exposure and DDIT4/ARHGAP15 as outcome

We used “FindMarkers” function to identify the input DEGs in CD4+ Memory T cells and acquired 67 genes. Functional enrichment analysis of input DEGs revealed the characterized groups in CD4+ Memory T cells, including T cell-related pathways, cellular response pathways, and inflammatory response pathways (Fig. S2C). eQTL exposures were obtained, and the GCA risk associated with the input DEGs was evaluated and visualized in Fig. 4A. Six genes with significant effects (IL32, ARHGAP15, RGCC, ARID5B, DDIT4, and TNFRSF25) were identified as significant exposures. DDIT4 and ARHGAP15 exhibited genetic risk effects for GCA, which were specifically focused on. The causal effects of ARHGAP15 in training dataset and validation dataset were visualized in Fig. 4B and Fig. S3A–D. ARHGAP15 showed no evidence of heterogeneity, with significant causal effect to GCA risk. The MR estimates of the whole 6 significant exposures in training and validation datasets were presented in Fig. 4C, D. In detail, DDIT4 exhibited the association with GCA risk with an OR of 5.1065 (95% CI 1.5281–17.0645) via Wald Ratio method, while ARHGAP15 was associated with the risk of GCA with an OR of 1.2622 (95% CI 1.0159–1.5683) calculated via IVW and 1.1917 (95% CI 0.8675–1.6372) calculated via MR-Egger in training dataset, and the results were further validated in validation dataset. Additionally, the results of test heterogeneity for all genes were presented in Table S1, and the horizontal pleiotropy test of all genes were presented in Table S2. Subsequently, to confirm the direction of causation between DDIT4/ARHGAP15 and GCA, reverse MR analyses were conducted (Fig. 4E). The result confirmed the causal role of DDIT4 and ARHGAP15 to GCA risk.

### Colocalization and phenoscanner database analysis

Colocalization analysis was conducted to further determine the probability that SNPs associated with disease and eQTL shared causal genetic variants. We conducted SNP-level colocalization analysis to evaluate the shared causal variants between the DDIT4/ARHGAP15 gene and GCA. Five hypotheses (H0–H4) were configured in the colocalization analysis for posterior probabilities calculation (Fig. S4A). The detailed potential associations between DDIT4/ARHGAP15 and GCA are shown in Fig. S4B, C. These associations involved rs11688752 (GCA-ARHGAP15) and rs11812792 (GCA-DDIT4). Phenoscanner was used to search for the diseases significantly associated with the SNP. Setting the p threshold as 5 × 10^–8^, we screened the associated phenotypes related to the SNPs of 6 identified causal genes using the database. The results showed that various diseases or phenotypes were associated with the SNP, including rheumatoid arthritis and ulcerative colitis, as described in Table S3.

### Significant genes in different cell clusters

**Fig. 5:**
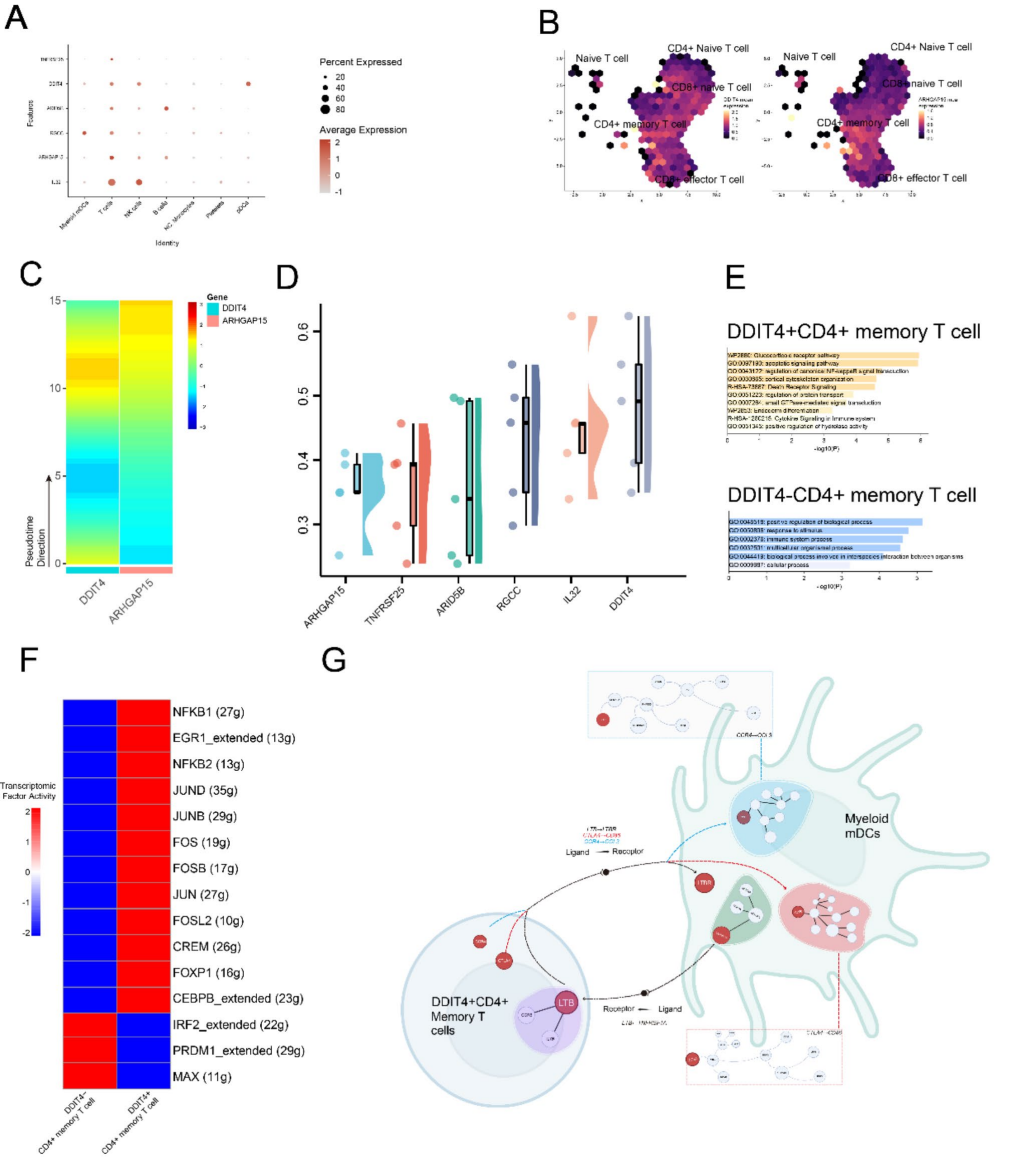
The single-cell analysis of significant genes in GCA samples. **A** The gene expression of IL32, ARHGAP15, RGCC, ARID5B, DDIT4 and TNFRSF25 in different types of cells in GCA. **B** The expression distribution of DDIT4 and ARHGAP15 in GCA. **C** Heatmap revealed the varied expression of DDIT4 and ARHGAP15 in CD4+ T cell differentiation trajectory in GCA. **D** The friend analysis of significant genes. **E** Functional enrichment of DEGs in DDIT4+ / − CD4+ Memory T cells, conducted by metascape. **F** Transcriptomic factor scores of DDIT4+ / − CD4+ Memory T cells in GCA. **G** Cell-specific activated communication pathways between CD4+ Memory T cells and myeloid mDCs, analyzed by CytoTalk

Using the “GeneSwitches” package, we identified the activate state of DEGs in CD4+ Memory T cells (Fig. S5A). The DEGs in CD4+ Memory T cells were mainly activated, while most surface proteins, as well as transcriptomic factors NME2 and NR1D1, were activated during the middle period of cell differentiation. After acquiring the significant genes by MR analysis, we visualized the expression of these genes in scRNA-seq GCA samples. All 6 genes showed noticeable expression in T cells, with DDIT4 and ARHGAP15 mainly expressed in CD4+ Memory T cells (Fig. 5A, B). Then we extracted and re-clustered 2000 random T cells from GCA samples, and subsequently “monocle” method was adopted to explore the expression variation of DDIT4 and ARHGAP15 at different stages of CD4+ Memory T cells in GCA (Fig. 5C). The expression trend of DDIT4 decreased in the early to middle stage, while it was upregulated in the later stage, corresponding to the differentiation of CD4+ Memory T cells. ARHGAP15 showed obvious expression in the terminal stage of differentiation. The friend analysis revealed the high correlation of DDIT4 with other 5 significant genes, while ARHGAP15 showed the strongest independence compared to other 5 genes (Fig. 5D). Considering the specifical expression of DDIT4 in cell distribution and time-related variation in CD4+ Memory T cells, and the high functional similarity of DDIT4 with other 5 genes, we speculated that DDIT4 exerts critical potential association with CD4+ Memory T cells in GCA, which was further comprehensively explored.

According to the expression or non-expression of DDIT4 and ARHGAP15, CD4+ Memory T cells were divided into DDIT4 ± or ARHGAP15 ± groups, respectively. The functional enrichment of DEGs identified from DDIT4 ± groups revealed the activity of apoptosis pathway and nuclear factor kappa-B (NF-κB) signal transduction in DDIT4+ CD4+ Memory T cells (Fig. 5E). SCENIC revealed the significant transcription factors in DDIT4 ± CD4+ Memory T cells (Fig. 5F). In DDIT4+ CD4+ Memory T cells, the transcriptomic factors related to immune response, T cell activation and cytokine production were upregulated, including JUN, NFKB, and FOS factor families. JUN and FOS transcriptomic factors are significant components of Activator Protein-1, which promotes inflammation mediator release and cytokine secretion. High intensity of NFKB1 and NFKB2 suggested high activation of NF-κB pathway in DDIT4+ Memory T cells in GCA.

Subsequently, through Cellchat analysis, compared with the non-expression group, both the DDIT4+ and ARHGAP15 + group showed stronger communication intensity and increased interactions with other cell types, especially through CD40 Ligand (CD40LG) ligand-receptors (Fig. S5B). DDIT4+ CD4+ Memory T cells groups showed high activity in IL16-CD4 ligand-receptor pair, while the DDIT4− group showed less activity (Fig. S5C). CD40LG and IL16-CD4 L-R pairs maintain inflammation response by enhancing the activation of APCs and the production of pro-inflammatory cytokines. Previous results from Cellchat revealed that DDIT4+ CD4+ T cells showed the strongest interactions with myeloid mDCs, we subsequently focused on the complex signaling networks between them using CytoTalk. LTB-LTBR, CTLA4-CD86, CCR4-CCL3, and TNFRSF1A-LTB ligand-receptor pairs were identified to be high-specific cell–cell communications between DDIT4+ CD4+ Memory T cells and myeloid mDCs (Fig. 5G). Lymphotoxin beta (LTB) is a potent chemokine that recruits myeloid cells to inflammation regions, inducing secretion of inflammation cytokines, such as CCR6 and IL7R. TNF Receptor Superfamily Member 1A (TNFRSF1A) activates the NF-κB, mitogen-activated protein kinase (MAPK), and other inflammation-related pathways to promote inflammation, while Cytotoxic T-Lymphocyte Associated Protein 4 (CTLA4) and C–C Motif Chemokine Receptor 4 (CCR4) regulate the overstimulated T cells and inhibit the immune response during inflammation. The variated incoming and outgoing signals in DDIT4+ CD4+ Memory T cells might suggest its special role of maintaining inflammation in GCA.

Based on the causal effect of DDIT4 and ARHGAP15 to GCA in CD4+ Memory T cells, we identified DEGs between DDIT4 ± and ARHGAP15 ± groups, and performed enrichment analysis using Metascape for each group (Fig. S5D). In DDIT4 ± groups, Metascape analysis indicated significant enrichment in pathways related to hemopoiesis, such as “regulation of hemopoiesis” and “hemopoiesis”; In ARHGAP15 ± groups, the T cells-related pathways were enriched, including “T cell receptor signaling pathway” and “T cell activation”.

### Causal proteins and metabolites of DDIT4+ CD4+ T cells in GCA

**Fig. 6:**
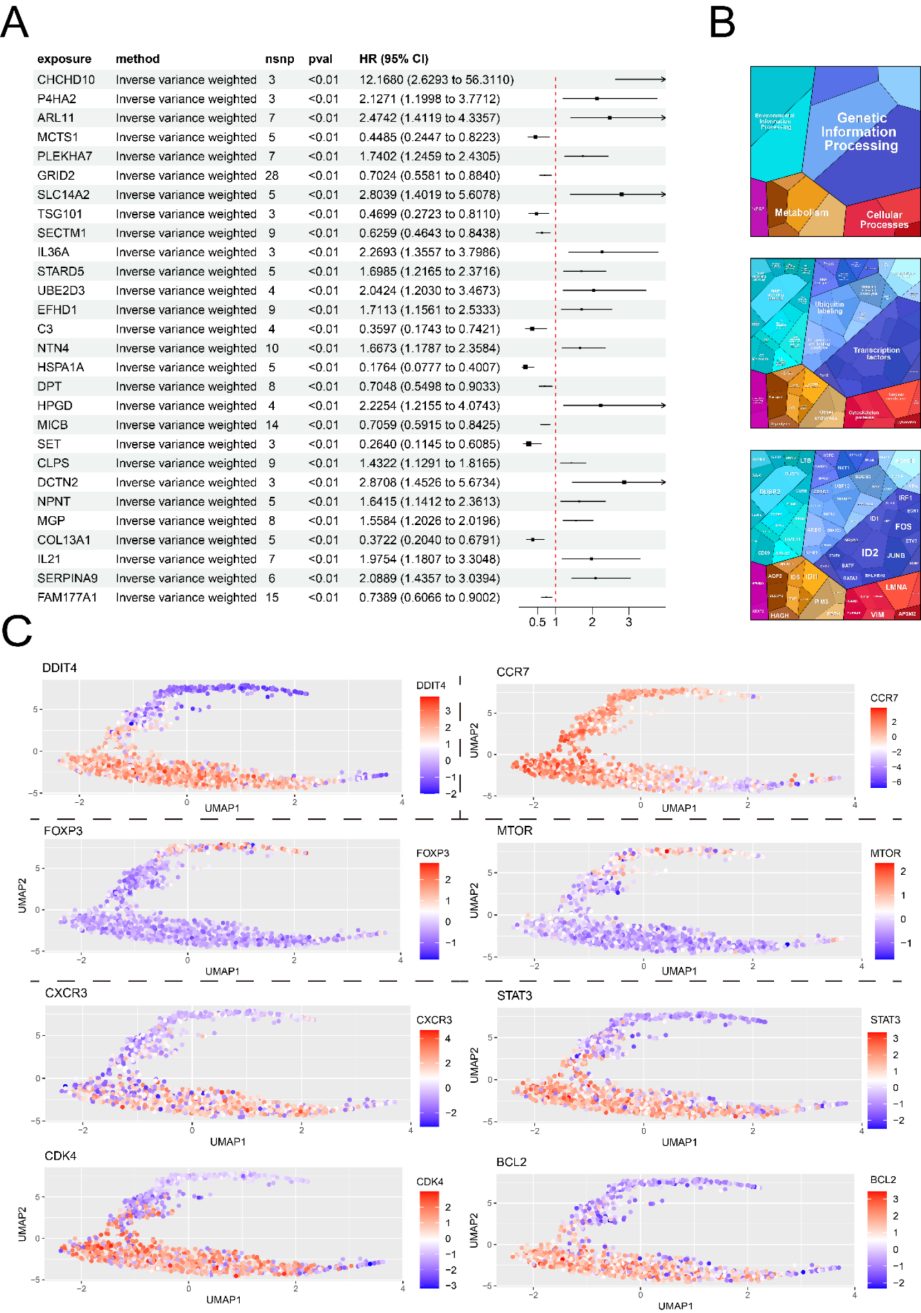
The pQTL MR analysis in GCA. **A** Causal plasma proteins related to GCA occurrence, estimated by IVW method, *p* < 0.01. **B** Enriched protein pathways of causal plasma protein, conducted by proteomaps. **C** Protein activities in DDIT4+ CD4+ Memory T cells

Through pQTL MR analysis, we discovered the plasma proteins, and biomarkers in blood and urine that show significant causal effects on GCA occurrence. 28 plasma proteins showed significant causal effect on GCA, with a threshold of *p* < 0.01 (Fig. 6A). Notably, several inflammation-related proteins were identified to be causal to GCA risk, especially IL36A and IL21. IL36A and IL21 actively participate in autoimmune disease, activating downstream pro-inflammatory pathways, thus promoting inflammatory responses. The Bonferroni adjusted results were showed in Fig. S6A. Figure 6B revealed the enriched pathways of causal proteins, which showed that various protein pathways participated in the occurrence and development of GCA, including genetic information pathway and transcriptional factors. SCENIC analysis results revealed the activated transcriptomic factors JUN and FOS, are essential components of Activate Protein-1, emphasized the promoted inflammation reaction in GCA. Moreover, we utilized “metaVIPER” method to evaluate the protein activity in DDIT4+ CD4+ Memory T cells (Fig. 6C). The co-expressed proteins, including CXCR3, CDK4, BCL2 and STAT3, mainly regulate the proliferation, differentiation and apoptosis of T cells, promoting the immune response in GCA. FOXP3 and MTOR inhibit the activity of T cells, inducing the phenotypes of Treg and maintaining suppression of immune responses. CCR7 expressed high activity in majority of DDIT4+ CD4+ Memory T cells, promoting the circulation of Memory T cells in blood. Different activated proteins revealed the potential approaches of inflammation maintained by DDIT4+ CD4+ Memory T cells in GCA.

**Fig. 7:**
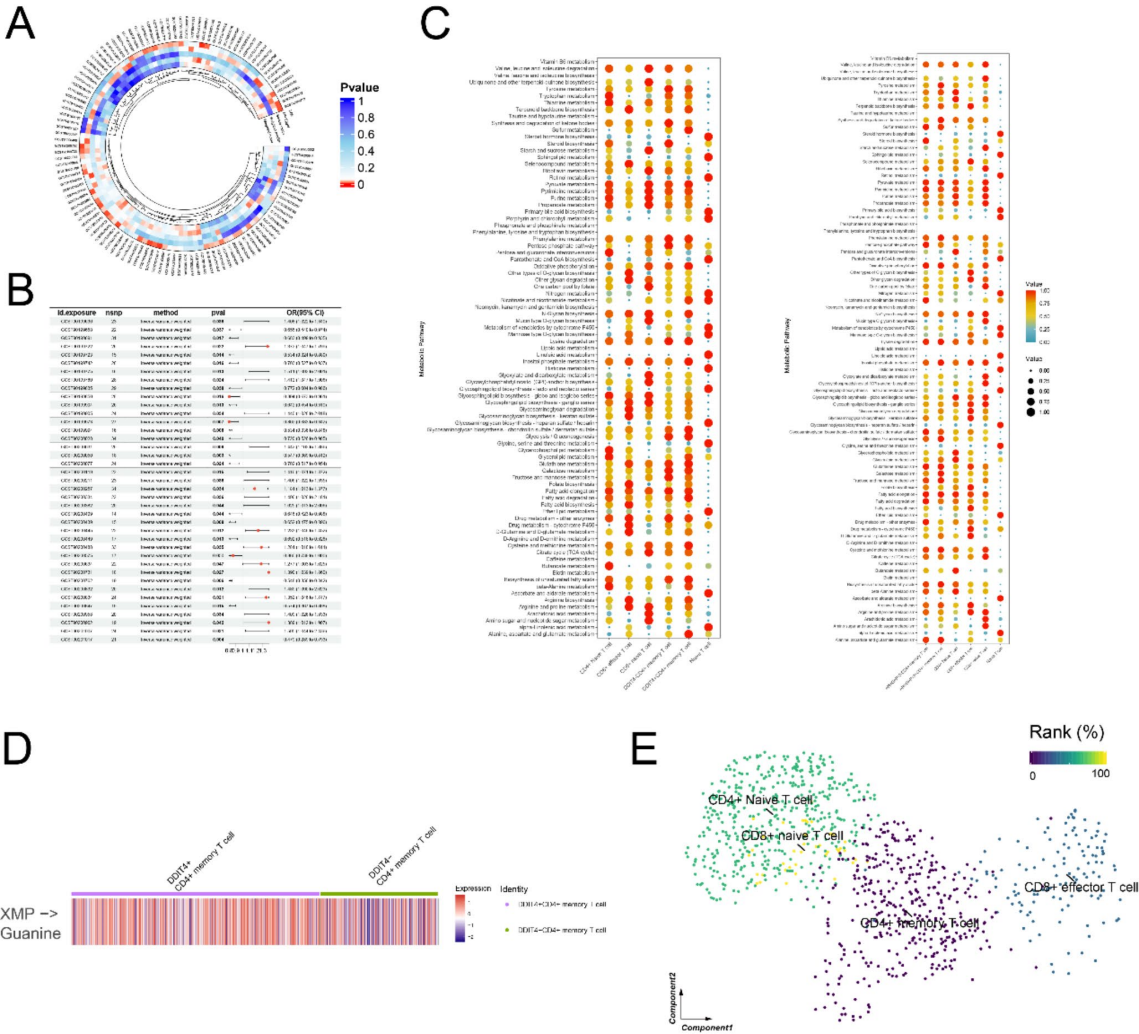
Metabolic analysis and drug sensitivity analysis of CD4+ Memory T cells in GCA. **A** The circle plot visualizes the significance of causal effect of each plasma metabolites on GCA. The concentric circles sequentially from the center represent weighted mode, weighted median, simple mode, MR-Egger and IVW. The colors indicate the significance of the effect. The redder, the more significant; The bluer, the less significant. **B** The MR estimates of significant plasma metabolites on GCA via IVW method. **C** Dotplots showing characterized metabolic pathways of DDIT4 ± and ARHGAP15 ± cell groups and other cell groups in T cells samples, both color and size indicate the effect size. **D** Metabolic flux of XMP to Guanine in DDIT4 ± CD4+ Memory T cells. **E** The drug response of different T subclusters,

Meanwhile, to explore the metabolism variation in GCA, we conducted the MR analysis to estimate the associations between metabolite levels and GCA risk (Fig. 7A, B). The results indicated that glutamine-related metabolites (GCST90200962), citrate-related metabolites (GCST90200832) and sucrose level (GCST90200449) have significant causal effects on GCA risk. Additionally, using “scMetabolism” package, we analyzed the metabolic pathway activities of DDIT4 ± and ARHGAP15 ± groups (Fig. 7C). Compared with DDIT4− group, DDIT4+ CD4+ Memory T cells were more metabolically active in the “Citrate cycle (TCA cycle)”, “Glycerolipid metabolism”, “D-Glutamine and D-glutamate metabolism”. The ARHGAP15 + group showed increased activities in “D-Glutamine and D-glutamate metabolism”, “Starch and sucrose metabolism”, and “Steroid biosynthesis” metabolic pathways. This may indicate the specific functional metabolic pathways of DDIT4 ± or the ARHGAP15 ± groups in GCA. Notably, DDIT4+ CD4+ Memory T cells exhibited elevated flux from xanthosine monophosphate (XMP) to Guanine, this metabolite pathway is essential for T cell proliferation. The high intensity of the metabolic process might suggest the upregulated proliferation of DDIT4+ CD4+ Memory T cells in GCA (Fig. 7D). Additionally, aspartate aminotransferase and C-reactive protein were identified as causal biomarkers in GCA (Fig. S6B, C).

Additionally, the drug response of different T cell subclusters in GCA revealed that the CD4+ Memory T cells showed the lowest response to DDIT4 inhibitory drugs, while CD4+ /CD8 + Naive T showed the highest, which further indicated the immune suppression of CD4+ Memory T cells (Fig. 7E).

### The regulation of cellular activity and lifespan of T cells by DDIT4

The GCA RNA-seq dataset GSE174694 was used to conduct validation on DDIT4. After dataset normalization, expression analysis between GCA and healthy control samples indicated that the expression of DDIT4 showed an obvious increase in GCA (Fig. S7A).

A genetically modified Jurkat cell line was employed to investigate the impact of DDIT4 on T cell signaling pathways. Concurrently, PMA was utilized to induce Jurkat into an activated state, which was then examined to ascertain the influence of DDIT4 on T cells in an activated state.

**Fig. 8:**
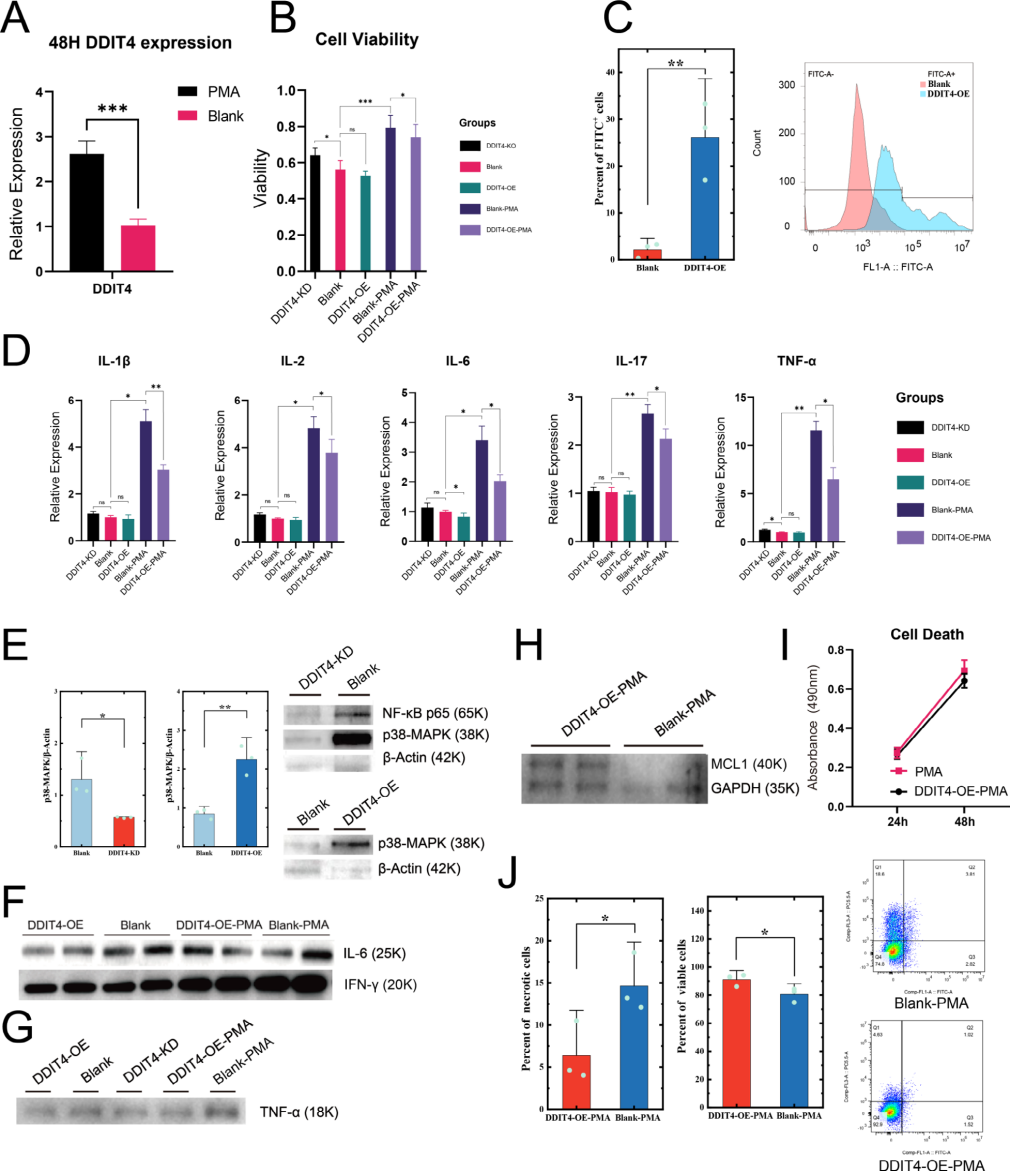
Comprehensive exploration of the regulation of DDIT4 on apoptosis of T cells. **A** The differential mRNA expression of DDIT4 in PMA-induced Jurkat cells and blank groups. **B** The cell viability of different groups of Jurkat cells, including DDIT4-KD/OE/Blank groups and the PMA induced/DDIT4-OE-PMA induced Jurkat cells. **C** The influence of DDIT4 on cell differentiation of T cells, evaluated by flow cytometry, including expression level of CD45 (X-axis), indicating the differentiation stages of Jurkat cells in DDIT4-OE and Blank groups (n = 3). **D** Detection of IL-1β, IL-2, IL-6, IL-17 and TNF-α expression levels in different groups of Jurkat cells (n = 3). **E** WB of NF-κB, p38-MAPK, β-Actin and their quantitative analysis in DDIT4-OE Jurkat cells, normal Jurkat cells and DDIT4-KD Jurkat cells. **F** WB of inflammatory factors INF-γ and IL-6 in culture of DDIT4-OE and PMA induced Jurkat cells. **G** WB of inflammatory factors TNF-α in culture of DDIT4-OE and PMA induced Jurkat cells. **H** WB of apoptosis-related protein MCL1 and GAPDH. **I** Examination of cytotoxicity of DDIT4 in PMA-induced groups. **J** The percentage of apoptotic cells in Blank-PMA and DDIT4-OE-PMA induced Jurkat cells, evaluated by flow cytometry analysis with Annexin V-FITC/PI staining (n = 3)

Jurkat cells were utilized to explore the specific function of DDIT4 in T cells. Overexpression of DDIT4 was achieved using the pIRES2-EGFP-DDIT4 plasmid, while knockdown was performed using the pKDL-DDIT4 plasmid. DDIT4 expression in the modified Jurkat cell lines was confirmed by Western blot analysis. (Fig. S10A). At the timepoint of 48 h after PMA induction, DDIT4 expression of PMA-induced group and Blank group was detected by qRT-PCR (Fig. 8A). PMA induced Jurkat activation, and a significant increase in DDIT4 expression.

CD4+ Memory T cells were individually extracted from single-cell samples for cell cycle analysis (Fig. S7B). Compared to the CT group, a greater proportion of CD4+ Memory T cells in GCA group were in S phase, which meant more cells were in preparation for the proliferation. Additionally, it also implies the avoiding of being exhausted of CD4+ Memory T cells in GCA progress. Considering the high expression of DDIT4 during CD4+ Memory T cell differentiation, we speculated that the cell state of CD4+ Memory T cells in GCA is regulated by DDIT4. To explore the effect of DDIT4 on Jurkat cells, we not only observed changes in the genetically modified Jurkat cells, but also assessed alterations in the activity following PMA induction as part of the experimental system. To determine the cellular activity of five Jurkat cells groups: DDIT4-KD, Blank, DDIT4-OE, Blank-PMA, and DDIT4-OE-PMA, CCK-8 assay was performed (Fig. 8B). Jurkat cells in the DDIT-KD group shows significant higher viability, DDIT4-OE group showed a slight lower in cellular activity. After PMA induction, the DDIT4-OE-PMA group also showed significant lower cellular activity compared to the Blank-PMA group. This implies that the upregulation of DDIT4 may prolong the lifespan of T cells by inhibiting their activities after adding PMA.

To further investigate the cell proliferation ability of DDIT4+ CD4+ Memory T cells, we also measured the proliferative capacity of the five groups of cells using the CCK-8 assay. Overexpression of DDIT4 inhibited the proliferation of Jurkat cells, whereas DDIT4-OE-PMA demonstrated lower proliferative ability than Blank-PMA after induction, which suggest that DDIT4 inhibit the cell proliferation of PMA-induced Jurkat cells (Fig. S8A). We use flow cytometry to examine the expression of CD45, a widely use marker for differentiation of Naive T cells into Memory T cells, of Jurkat cells in DDIT4-OE and Blank groups (Fig. 8C, S8B–D). DDIT4-OE group showed an obvious tendency to differentiate into Memory T cells compared with the Blank group. Overexpression of DDIT-4 promoted the phenotypic upregulation of memory T cells.

To further explore the immune functions of DDIT4+ T cells, we examined the expression of cytokines of DDIT4-KD, Blank, DDIT4-OE, Blank-PMA, and DDIT4-OE-PMA groups. There was no significant difference in IL-1β, IL-2, and IL-17 expression among the DDIT4-KD, Blank, and DDIT4-OE groups (Fig. 8D). IL-6 expression has downregulations in the DDIT4-OE group and TNF-α expression has upregulations in the DDIT4-KD group. In contrast, after the intervention of PMA, the expression of these inflammatory factors in both Blank-PMA, DDIT4-OE-PMA groups increased significantly, compared with groups which were not treated with PMA. Notably, the expression of all these inflammatory factors in DDIT4-OE-PMA were significantly lower than Blank-PMA group, which might indicate that DDIT4 triggers low-intensity inflammation in active T cells.

Bioinformatic analysis revealed that NF-κB/MAPK pathways were closely related to DDIT4+ Memory T cells. To validate the regulation of DDIT4 to the pathways, the Western blotting assay was conducted. Results showed that the overexpression of DDIT4 significantly upregulated the expression of NF-κB and MAPK, while the KD group showed the decreased expression of pathway proteins (Fig. 8E, S10B, C). The expression of IFN-γ, TNF-α and IL-6 in the serum-free culture medium of DDIT4-OE/Blank and DDIT4-OE/Blank-PMA groups showed that after PMA induction, the secretion of inflammatory factors increased, while DDIT4 overexpression slightly inhibited this upregulation trend (Fig. 8F, G). Combined with the low mTOR protein activity of the highly active subpopulation of DDIT4 mentioned above (Fig. 6C), we suggest that DDIT4 causes only a limited increase in T-cell activity after antigen stimulation.

Given that CD4+ Memory T cells showed a declined proportion of active cells (Fig. S7B), we hypothesized that high expression of DDIT4 inhibits the activation and regulates the lifespan of CD4+ Memory T cells. Myeloid Cell Leukemia 1 (MCL1) is an anti-apoptotic protein which extends the lifespan of cells. Although the previous results revealed that BCL2, a DDIT4+ memory T cell in the GCA group, exhibited heightened protein activity, given that BCL2 is regulated by TNF-α and DDIT4 influences the expression of TNF-α, we elected to examine MCL1, which performs a comparable function to BCL2, to elucidate the impact of DDIT4 on apoptosis. We detected expression in 5 Jurkat cell groups by Western blot method. DDIT4 upregulation promotes the upregulation of MCL1 in Jurkat cells (Fig. 8H, S10D). The cytotoxicity of DDIT4 expression was also investigated via LDHB assay. Compared with the Blank-PMA group, the DDIT4-OE-PMA group showed lower cell death, suggesting that DDIT4 regulates PMA-induced Jurkat cells (Fig. 8I). The proportions of apoptotic cells in Blank-PMA and DDIT4-OE-PMA induced Jurkat cells were evaluated by flow cytometry, which revealed that DDIT4 overexpression inhibited the apoptosis of PMA induced Jurkat cells (Fig. 8J, S9A–C). The results of the apoptosis and cytotoxicity assays indicated that Jurkat cells with high expression of DDIT4 even after PMA stimulation could maintain a longer cell life. The altered cell lifespan, cellular activity and inflammatory factor secretion suggested a chronic and low-intensity inflammatory state of DDIT4+ CD4+ Memory T cells.

To confirm the diagnostic significance of DDIT4 and ARHGAP15 for GCA, we evaluated the area under the curve (AUC) of the diagnostic receiver operating characteristic (ROC). DDIT4 and ARHGAP15 showed high accuracy in detecting GCA, with the AUC of the diagnostic ROC for the two genes reaching 0.819 and 0.884, respectively (Fig. S7C). Typical markers CFP and ESR1 achieved the AUCs of 0.537 and 0.882 respectively (Fig. S7D).

## Discussion

Giant cell arteritis is currently the most prevalent primary systemic vasculitis among patients over 50 [49]. If untreated, the risk of serious complications increases, including blindness, aortic aneurysm and dissection [50]. Thus, rapid diagnosis and pathogen identification of the disease are essential for better prevention and treatment. Via single-cell analysis and MR analysis, we comprehensively explored the changes in CD4+ Memory T cells in GCA and investigated the roles of two significant genes, DDIT4 and ARHGAP15, in the progression of the disease. Furthermore, the potential involvement of DDIT4 in disease pathogenesis was investigated in great detail.

Single-cell analysis revealed the proportions of main cell types in GCA. T cells showed an obvious decrease in GCA and samples from the elderly, compared with the controls. Subsequently T cell clusters were extracted and re-clustered, and an obvious increase of CD4+ T cells was observed, particularly CD4+ Memory T cells. The scPagwas analysis indicated the pathogenetic cell clusters of GCA was CD4+ Naive T cells, and the perturbation of Naive T cells was bigger than other types of cells in GCA and aging.

Aging weakens blood vessels, which could lead to inflammation-related vascular lesions [51]. Additionally, aging promotes immune system restructuring and variations in immune-related cell proportions, typically resulting in decreased host protection and increased tissue inflammation in autoimmune diseases [52]. Specifically, CD4+ T cells are triggered by class-II major histocompatibility complex (MHC-II) and co-stimulatory molecules (CD80 and CD86), then infiltrated all the layers of the artery, leading to pan-arteritis [53, 54]. Given that the CD4+ Memory T cells experienced smallest perturbation in GCA and the old, it can be assumed that compared with short-lived cells like effector cells, CD4+ Memory T cells might persist longer beyond antigen clearance, and mount a quicker, higher-magnitude anamnestic response, potentially playing a more pivotal role in GCA [55]. Additionally, memory T cells are crucial for maintaining chronic inflammation.

GCA is more prevalent in the elderly, which might indicate potential associations between CD4+ Memory T cells in GCA and aging. Similarities of CD4+ Memory T cells in GCA and old samples were further explored. Using Jaccard method, we compared the similarity of different T cell clusters in two groups, and identified the similar genes and functional pathways in CD4+ Memory T cells. Results indicated that CD4+ Memory T cells in GCA and old samples showed similar enriched pathways, including actin filament regulation, cell–cell adhesion, focal adhesion and actin binding. During the aging process, the dysregulation in actin cytoskeleton function can lead to a decline in T cell function, declining immune response; in inflammation, actin binding proteins regulate the activation and migration of T cells, participating in the amplification and development of inflammation [56]. CD4+ Memory T cells in T cell trajectory differentiate during the middle-late phase in both GCA and old samples, which might be attributed to the myeloid-endothelial-CD4+ T cell hierarchical response. Notably, CD4+ Memory T cells showed an individual differentiation branch. Additionally, CD4+ T cells in the elderly express similar communication patterns with those in GCA, mainly communicating with myeloids via various MIF pathways. Macrophage migration inhibitory factor (MIF) is significant in pro-inflammatory reactions by interacting with chemokines receptors [57, 58], promoting immune-cell recruitment and macrophage activation for further inflammatory response. Similar functional pathways, communications and cell differentiation stage of CD4+ Memory T cells suggest the common characteristics, including pro-inflammatory reactions, and weaker T cell functions, while the different cell differentiation branch and communication pathways indicated the slight differences between CD4+ Memory T cells in GCA and aging process.

Focusing on CD4+ Memory T cells, we identified differentially expressed genes and assessed their risk using MR design. DDIT4 and ARHGAP15 were evaluated as significant risks for GCA patients, with DDIT4 showing a relatively higher risk. Several studies have reported that DDIT4 extended the lifespan of cells by inhibiting mTORC1. The overexpressed DDIT4 promotes the development of various cancers [59–61], as well as aggravates the vascular injury in inflammation response [62]. Parallelly, the reduced inflammation resulted from DDIT4 knockdown was related to NF-κB signaling pathway [63]. The C-terminal region of DDIT4 can interact with and sequestered IκBα, which continuously activates pro-inflammatory NF-κB signaling pathway after inflammation stimulations [63]. The pro-inflammatory effect of DDIT4 sheds light on the potential inflammation mechanism in GCA, and the long-lived and rapid-antigen reacting CD4+ Memory T cells might amplify the function of DDIT4, and eventually lead to the vascular inflammation dysregulation and GCA. ARHGAP15 is a regulator of Rac1. It has specific GAP (enzyme activating protein) activity in Rac1, which negatively regulates Rac1 by promoting GTP hydrolysis [64]. In the inflammatory environment of GCA, the increase of ARHGAP15 might lead to the decreased intensity of Rac1, and the dysregulated cell adhesion caused abnormal infiltration and death of CD4+ T cells in vessel walls as well as development of GCA.

After identifying DDIT4 and ARHGAP15, we divided the CD4+ Memory T cells in GCA samples into DDIT4 ± and ARHGAP15 ± groups based on their expression. Then the differences between DDIT4 ± CD4+ Memory T cells were explored. Functional enrichment identified differential pathways in DDIT4+ and DDIT4− groups. In DDIT4+ group, the apoptosis and NF-κB signaling pathway were more activated. Notably, the subsequent transcriptomic factor analysis and communication analysis further highlighted the NF-κB signaling pathway. NFKB1 and NFKB2 transcriptomic factors showed high intensity in DDIT4+ CD4+ Memory T cells. CytoTalk communication analysis indicated the significant cell signals through TNFRSF1A, which activates the NF-κB, MAPK and other inflammation related pathways to promote the inflammation. Generally, high expression of DDIT4 indicated that the upregulated inflammation response and NF-κB signaling transduction pathway are involved in molecular mechanisms of GCA development.

Apart from eQTL analysis, we also performed MR analysis on protein and metabolite levels. The functions of causal proteins associated to GCA mainly lie in transcriptomic factor, glycolysis metabolism, cytoskeleton establishment and different signaling pathways. Notably, NF-κB and MAPK pathways were significantly enriched in protein pathways. The co-expressed proteins, including CXCR3, CDK4, BCL2 and STAT3, promote the proliferation of T cells, inhibit the apoptosis and induce the differentiation of T cells to Memory cells; the non-coexpressed FOXP3 and mTOR mainly inhibit the activity of T cells through Treg induction and metabolic regulation. pQTL analysis, revealed that the occurrence and development of GCA are the integrated results of various factors, including gene regulation, transcriptomic factor, signaling pathway and protein activities, with close associations with NF-κB pathway. Additionally, the significantly differentially activated metabolic pathways between DDIT4 ± and ARHGAP15 ± groups mainly involve the metabolism of glutamine, the citrate cycle, and sucrose levels. During immune responses to inflammation, T cells are often induced to express excessive metabolic and cellular respiration levels [65]. Glutamate metabolism maintains the critical TCA cycle in response to glucose deprivation or reduced pyruvate supply by catabolizing glutamine into alpha-ketoglutarate (α-KG) to replenish TCA cycle intermediates and sustain mitochondrial respiration [66, 67], which was completed by aspartate aminotransferase (AST). The causal effect of AST to GCA evidenced the maintenance of glutamate metabolism to TCA cycle. Since glutamine depletion blocks proliferation and cytokine production, T cell activation selectively increases glutamine uptake [68]. In GCA, DDIT4+ /ARHGAP15 + CD4+ Memory T cells exhibit active cell respiration and metabolism by promoting citrate circle or sucrose metabolism, while the over-activated cell respiration might result in cell overload, oxidative stress, immune exhaustion and early apoptosis eventually, aggravating vascular inflammation. The aging immune system is fragile, and continuous over-activated cell respiration can cause more severe cell overload and chronic inflammation, which are typical characteristics of GCA.

Since DDIT4 showed relatively higher causal risk to GCA occurrence, the in vitro experiments were conducted on Jurkat cells to explore the specific function of DDIT4. Experiment results indicated that DDIT4 regulates the cellular activity of T cells, and promotes the differentiation of Memory T cells. Moreover, DDIT4+ Memory T cells have a longer lifespan and also maintain high response properties, compared with other groups of cells. Considering the high intensity in XMP to Guanine metabolite flux in DDIT4+ CD4+ Memory T cells, and low response to drugs of CD4+ Memory T cells, this phenotype may reduce overall level of inflammation, but the duration of inflammation may be prolonged, leading to a chronic, low-intensity inflammatory state in patients. Since GCA is characterized as the sustained inflammation response with relative low extent, the characteristic might be associated with the chronic inflammatory state triggered by DDIT4.

However, there are some limitations that should be taken into consideration when evaluating the results. First, though we comprehensively explored CD4+ Memory T cells in GCA and identified two significant genes, DDIT4 and ARHGAP15, the overall effect of two genes on GCA was unable to be evaluated. DDIT4 and ARHGAP15 may regulate GCA progress via various mechanisms, including macrophages, smooth muscle cells and PERK/ATF4 pathway [69–71]. Second, our MR analysis was based on European populations, which may limit the generalizability of our results. Lastly, the gender or sex were not taken into analysis. Despite these limitations, our study is the first to explore significant genes in CD4+ Memory T cells that may have causal links to GCA.

## Conclusion

In summary, our research identified two significant risk genes, DDIT4 and ARHGAP15, in GCA. By integrating transcriptomic and MR analyses, we provided in-depth insights into significant genes in CD4+ Memory T cells in GCA and identified causal effects between DDIT4/ARHGAP15 and the occurrence of GCA. After identifying DDIT4 and ARHGAP15, we further explored their regulatory effects on CD4+ Memory T cells in GCA, including cell differentiation, functional pathways, intercellular communications, protein activity and metabolism. Moreover, experiments conducted on Jurkat cell lines showed that DDIT4 maintains inflammation via prolonging the T cell lifespan and inhibiting cellular activity, triggering continuous and chronic inflammatory response, eventually promoting the progression of GCA. Our work may help clarify the function of CD4+ Memory T cells in GCA, and provide new perspectives for pathogen elucidation and therapeutic targets.

## Electronic supplementary material

Below is the link to the electronic supplementary material.

Supplementary Material 1

Supplementary Material 2

## Data Availability

The datasets selected in our research can be found and downloaded for free online, while the databases we searched and accession number(s) are all provided in article or supplement materials.

## Abbreviations

GCA: Giant cell arteritis
MR: Mendelian randomization
SNPs: Single nucleotide polymorphisms
GEO: Gene Expression Omnibus
FFPE: Formalin-fixed paraffin-embedded
eQTL: Expression quantitative trait loci
GWAS: Genome-wide association studies
CLSA: Canadian Longitudinal Study on Aging
PCA: Principal component analysis
UMAP: Uniform Manifold Approximation and Projection
MST: Minimum spanning tree
DEG: Differentially expressed gene
LD: Linkage disequilibrium
IVW: Inverse variance weighted
PheWAS: Phenome-wide association study
ROC: Receiver Operation Characteristic
mDCs: Myeloid dendritic cells
NC Monocytes: Non-classical monocytes
pDC: Plasmacytoid dendritic cells
AUC: Area Under Curve
FBS: Fetal bovine growth serum
qRT-PCR: Quantitative Real-time Polymerase Chain Reaction
CCK-8: Cell Counting Kit-8
LDH: Lactate dehydrogenase
MHC-II: Class-II major histocompatibility complex
MIF: Macrophage migration inhibitory factor
mTORC1: Mammalian target of rapamycin complex
Rac1: Ras-related C3 botulinum toxin substrate 1
α-KG: Alpha-ketoglutarate. AST: aspartate aminotransferase

## References

[1] Pugh D, Karabayas M, Basu N, Cid MC, Goel R, Goodyear CS, Grayson PC, McAdoo SP, Mason JC, Owen C, et al. Large-vessel vasculitis. Nat Rev Dis Prim. 2022;7(1):93. 34992251 10.1038/s41572-021-00327-5 PMC9115766

[2] Crowson CS, Matteson EL, Myasoedova E, Michet CJ, Ernste FC, Warrington KJ, Davis JM 3rd, Hunder GG, Therneau TM, Gabriel SE. The lifetime risk of adult-onset rheumatoid arthritis and other inflammatory autoimmune rheumatic diseases. Arthritis Rheum. 2011;63(3):633–9. 21360492 10.1002/art.30155 PMC3078757

[3] Monti S, Milanesi A, Klersy C, Tomelleri A, Dagna L, Campochiaro C, Farina N, Muratore F, Galli E, Marvisi C, et al. Age at diagnosis influences the clinical phenotype, treatment strategies and outcomes in patients with giant cell arteritis: results from the observational GCAGE study on a large cohort of 1004 patients. Ann Rheum Dis. 2023;82(8):1098–106. 37188498 10.1136/ard-2023-223895

[4] Stamatis P, Turesson C, Willim M, Nilsson J, Englund M, Mohammad AJ. Malignancies in giant cell arteritis: a population-based cohort study. J Rheumatol. 2020;47(3):400–6. 31154410 10.3899/jrheum.190236

[5] Mackie SL, Dasgupta B. Vasculitis syndromes: dealing with increased vascular risk and mortality in GCA. Nat Rev Rheumatol. 2014;10(5):264–5. 24637366 10.1038/nrrheum.2014.38

[6] Lyons HS, Quick V, Sinclair AJ, Nagaraju S, Mollan SP. A new era for giant cell arteritis. Eye (Lond). 2020;34(6):1013–26. 31582795 10.1038/s41433-019-0608-7 PMC7253415

[7] Ponte C, Martins-Martinho J, Luqmani RA. Diagnosis of giant cell arteritis. Rheumatology (Oxford). 2020;59(Suppl 3):iii5–16. 32348512 10.1093/rheumatology/kez553

[8] Vieira M, Régnier P, Maciejewski-Duval A, Le Joncour A, Darasse-Jèze G, Rosenzwajg M, Klatzmann D, Cacoub P, Saadoun D. Interferon signature in giant cell arteritis aortitis. J Autoimmun. 2022;127: 102796. 35123212 10.1016/j.jaut.2022.102796

[9] Akiyama M, Ohtsuki S, Berry GJ, Liang DH, Goronzy JJ, Weyand CM. Innate and adaptive immunity in giant cell arteritis. Front Immunol. 2020;11: 621098. 33717054 10.3389/fimmu.2020.621098 PMC7947610

[10] Whitney ML, Jefferson LS, Kimball SR. ATF4 is necessary and sufficient for ER stress-induced upregulation of REDD1 expression. Biochem Biophys Res Commun. 2009;379(2):451–5. 19114033 10.1016/j.bbrc.2008.12.079 PMC2656673

[11] Reuschel EL, Wang J, Shivers DK, Muthumani K, Weiner DB, Ma Z, Finkel TH. REDD1 is essential for optimal T cell proliferation and survival. PLoS ONE. 2015;10(8): e0136323. 26301899 10.1371/journal.pone.0136323 PMC4547781

[12] Yang C-A, Li J-P, Lai Y-H, Huang Y-L, Lin C-Y, Lan J-L. Assessing the immune cell subset and genetic mutations in patients with palindromic rheumatism seronegative for rheumatoid factor and anti-cyclic citrullinated peptide. Arthritis Rheumatol. 2023;75(2):187–200. 35819819 10.1002/art.42297

[13] Zhidkova EM, Lylova ES, Grigoreva DD, Kirsanov KI, Osipova AV, Kulikov EP, Mertsalov SA, Belitsky GA, Budunova I, Yakubovskaya MG, et al. Nutritional Sensor REDD1 in cancer and inflammation: friend or foe? Int J Mol Sci. 2022;23(17):9686. 36077083 10.3390/ijms23179686 PMC9456073

[14] Vega-Rubin-de-Celis S, Abdallah Z, Kinch L, Grishin NV, Brugarolas J, Zhang X. Structural analysis and functional implications of the negative mTORC1 regulator REDD1. Biochemistry. 2010;49(11):2491–501. 20166753 10.1021/bi902135e PMC3046781

[15] Lipina C, Hundal HS. Is REDD1 a metabolic éminence grise? Trends Endocrinol Metab. 2016;27(12):868–80. 27613400 10.1016/j.tem.2016.08.005 PMC5119498

[16] Costa C, Germena G, Martin-Conte EL, Molineris I, Bosco E, Marengo S, Azzolino O, Altruda F, Ranieri VM, Hirsch E. The RacGAP ArhGAP15 is a master negative regulator of neutrophil functions. Blood. 2011;118(4):1099–108. 21551229 10.1182/blood-2010-12-324756

[17] Fanzo JC, Yang W, Jang SY, Gupta S, Chen Q, Siddiq A, Greenberg S, Pernis AB. Loss of IRF-4-binding protein leads to the spontaneous development of systemic autoimmunity. J Clin Investig. 2006;116(3):703–14. 16470246 10.1172/JCI24096 PMC1361345

[18] Luo OJ, Lei W, Zhu G, Ren Z, Xu Y, Xiao C, Zhang H, Cai J, Luo Z, Gao L, et al. Multidimensional single-cell analysis of human peripheral blood reveals characteristic features of the immune system landscape in aging and frailty. Nat Aging. 2022;2(4):348–64. 37117750 10.1038/s43587-022-00198-9

[19] Reitsema RD, van der Geest KSM, Sandovici M, Jiemy WF, Graver JC, Abdulahad WH, Boots AMH, Heeringa P, Brouwer E. Phenotypic, transcriptomic and functional profiling reveal reduced activation thresholds of CD8+ T cells in giant cell arteritis. Rheumatology (Oxford). 2022;62(1):417–27. 35460236 10.1093/rheumatology/keac250 PMC9788826

[20] Rust R, Grönnert L, Weber RZ, Mulders G, Schwab ME. Refueling the ischemic CNS: guidance molecules for vascular repair. Trends Neurosci. 2019;42(9):644–56. 31285047 10.1016/j.tins.2019.05.006

[21] Chen Y, Lu T, Pettersson-Kymmer U, Stewart ID, Butler-Laporte G, Nakanishi T, Cerani A, Liang KYH, Yoshiji S, Willett JDS, et al. Genomic atlas of the plasma metabolome prioritizes metabolites implicated in human diseases. Nat Genet. 2023;55(1):44–53. 36635386 10.1038/s41588-022-01270-1 PMC7614162

[22] Sinnott-Armstrong N, Tanigawa Y, Amar D, Mars N, Benner C, Aguirre M, Venkataraman GR, Wainberg M, Ollila HM, Kiiskinen T, et al. Genetics of 35 blood and urine biomarkers in the UK Biobank. Nat Genet. 2021;53(2):185–94. 33462484 10.1038/s41588-020-00757-z PMC7867639

[23] Hao Y, Hao S, Andersen-Nissen E, Mauck WM 3rd, Zheng S, Butler A, Lee MJ, Wilk AJ, Darby C, Zager M, et al. Integrated analysis of multimodal single-cell data. Cell. 2021;184(13):3573-3587.e3529. 34062119 10.1016/j.cell.2021.04.048 PMC8238499

[24] Korsunsky I, Millard N, Fan J, Slowikowski K, Zhang F, Wei K, Baglaenko Y, Brenner M, Loh PR, Raychaudhuri S. Fast, sensitive and accurate integration of single-cell data with Harmony. Nat Methods. 2019;16(12):1289–96. 31740819 10.1038/s41592-019-0619-0 PMC6884693

[25] Skinnider MA, Squair JW, Kathe C, Anderson MA, Gautier M, Matson KJE, Milano M, Hutson TH, Barraud Q, Phillips AA, et al. Cell type prioritization in single-cell data. Nat Biotechnol. 2021;39(1):30–4. 32690972 10.1038/s41587-020-0605-1 PMC7610525

[26] Liu Q, Wang Z, Jiang Y, Shao F, Ma Y, Zhu M, Luo Q, Bi Y, Cao L, Peng L, et al. Single-cell landscape analysis reveals distinct regression trajectories and novel prognostic biomarkers in primary neuroblastoma. Genes Dis. 2022;9(6):1624–38. 36157484 10.1016/j.gendis.2021.12.020 PMC9485279

[27] Street K, Risso D, Fletcher RB, Das D, Ngai J, Yosef N, Purdom E, Dudoit S. Slingshot: cell lineage and pseudotime inference for single-cell transcriptomics. BMC Genom. 2018;19(1):477. 10.1186/s12864-018-4772-0 PMC6007078 29914354

[28] Trapnell C, Cacchiarelli D, Grimsby J, Pokharel P, Li S, Morse M, Lennon NJ, Livak KJ, Mikkelsen TS, Rinn JL. The dynamics and regulators of cell fate decisions are revealed by pseudotemporal ordering of single cells. Nat Biotechnol. 2014;32(4):381–6. 24658644 10.1038/nbt.2859 PMC4122333

[29] Jin S, Guerrero-Juarez CF, Zhang L, Chang I, Ramos R, Kuan CH, Myung P, Plikus MV, Nie Q. Inference and analysis of cell-cell communication using cell chat. Nat Commun. 2021;12(1):1088. 33597522 10.1038/s41467-021-21246-9 PMC7889871

[30] Hemani G, Zheng J, Elsworth B, Wade KH, Haberland V, Baird D, Laurin C, Burgess S, Bowden J, Langdon R, et al. The MR-Base platform supports systematic causal inference across the human phenome. Life. 2018;7:e34408. 10.7554/eLife.34408 PMC5976434 29846171

[31] Abecasis GR, Auton A, Brooks LD, DePristo MA, Durbin RM, Handsaker RE, Kang HM, Marth GT, McVean GA. An integrated map of genetic variation from 1,092 human genomes. Nature. 2012;491(7422):56–65. 23128226 10.1038/nature11632 PMC3498066

[32] Wang L, Zhou L, ZhangBao J, Huang W, Tan H, Fan Y, Lu C, Yu J, Wang M, Lu J, et al. Causal associations between prodromal infection and neuromyelitis optica spectrum disorder: a Mendelian randomization study. Eur J Neurol. 2023;30(12):3819–27. 37540821 10.1111/ene.16014

[33] Duan L, Xiao R, Liu S, Shi Y, Feng Y. Causality between cognitive performance and cardiovascular disease: a bidirectional Mendelian randomization study. Gene. 2024;891: 147822. 37758004 10.1016/j.gene.2023.147822

[34] Ding H, Douglass EF Jr, Sonabend AM, Mela A, Bose S, Gonzalez C, Canoll PD, Sims PA, Alvarez MJ, Califano A. Quantitative assessment of protein activity in orphan tissues and single cells using the metaVIPER algorithm. Nat Commun. 2018;9(1):1471. 29662057 10.1038/s41467-018-03843-3 PMC5902599

[35] Alvarez MJ, Shen Y, Giorgi FM, Lachmann A, Ding BB, Ye BH, Califano A. Functional characterization of somatic mutations in cancer using network-based inference of protein activity. Nat Genet. 2016;48(8):838–47. 27322546 10.1038/ng.3593 PMC5040167

[36] Margolin AA, Nemenman I, Basso K, Wiggins C, Stolovitzky G, Favera RD, Califano A: ARACNE: an algorithm for the reconstruction of gene regulatory networks in a mammalian cellular context. In: BMC bioinformatics.2006: Springer. 2006. pp. 1–15. 10.1186/1471-2105-7-S1-S7 PMC1810318 16723010

[37] Wang G, Sarkar A, Carbonetto P, Stephens M. A simple new approach to variable selection in regression, with application to genetic fine mapping. J R Stat Soc Ser B Stat Methodol. 2020;82(5):1273–300. 10.1111/rssb.12388 PMC10201948 37220626

[38] Kamat MA, Blackshaw JA, Young R, Surendran P, Burgess S, Danesh J, Butterworth AS, Staley JR. PhenoScanner V2: an expanded tool for searching human genotype-phenotype associations. Bioinformatics (Oxford, England). 2019;35(22):4851–3. 31233103 10.1093/bioinformatics/btz469 PMC6853652

[39] Ye X, Liu B, Bai Y, Cao Y, Lin S, Lyu L, Meng H, Dai Y, Ye D, Pan W, et al. Genetic evidence strengthens the bidirectional connection between gut microbiota and periodontitis: insights from a two-sample Mendelian randomization study. J Transl Med. 2023;21(1):674. 37770955 10.1186/s12967-023-04559-9 PMC10537583

[40] Cao EY, Ouyang JF, Rackham OJ. GeneSwitches: ordering gene expression and functional events in single-cell experiments. Bioinformatics. 2020;36(10):3273–5. 32058565 10.1093/bioinformatics/btaa099

[41] Aibar S, González-Blas CB, Moerman T, Huynh-Thu VA, Imrichova H, Hulselmans G, Rambow F, Marine J-C, Geurts P, Aerts J. SCENIC: single-cell regulatory network inference and clustering. Nat Methods. 2017;14(11):1083–6. 28991892 10.1038/nmeth.4463 PMC5937676

[42] Hu Y, Peng T, Gao L, Tan K. CytoTalk: De novo construction of signal transduction networks using single-cell transcriptomic data. Sci Adv. 2021;7(16):eabf1356. 33853780 10.1126/sciadv.abf1356 PMC8046375

[43] Zheng SC, Stein-O’Brien G, Augustin JJ, Slosberg J, Carosso GA, Winer B, Shin G, Bjornsson HT, Goff LA, Hansen KD. Universal prediction of cell-cycle position using transfer learning. Genome Biol. 2022;23(1):41. 35101061 10.1186/s13059-021-02581-y PMC8802487

[44] Wu Y, Yang S, Ma J, Chen Z, Song G, Rao D, Cheng Y, Huang S, Liu Y, Jiang S, et al. Spatiotemporal immune landscape of colorectal cancer liver metastasis at single-cell level. Cancer Discov. 2022;12(1):134–53. 34417225 10.1158/2159-8290.CD-21-0316

[45] Alghamdi N, Chang W, Dang P, Lu X, Wan C, Gampala S, Huang Z, Wang J, Ma Q, Zang Y. A graph neural network model to estimate cell-wise metabolic flux using single-cell RNA-seq data. Genome Res. 2021;31(10):1867–84. 34301623 10.1101/gr.271205.120 PMC8494226

[46] Li C, Shao X, Zhang S, Wang Y, Jin K, Yang P, Lu X, Fan X, Wang Y. scRank infers drug-responsive cell types from untreated scRNA-seq data using a target-perturbed gene regulatory network. Cell Rep Med. 2024;5(6):101568. 38754419 10.1016/j.xcrm.2024.101568 PMC11228399

[47] Chan FLY, Lester S, Whittle SL, Hill CL. The utility of ESR, CRP and platelets in the diagnosis of GCA. BMC Rheumatol. 2019;3:14. 31008443 10.1186/s41927-019-0061-z PMC6456976

[48] Yu G, Li F, Qin Y, Bo X, Wu Y, Wang S. GOSemSim: an R package for measuring semantic similarity among GO terms and gene products. Bioinformatics (Oxford, England). 2010;26(7):976–8. 20179076 10.1093/bioinformatics/btq064

[49] Chehem Daoud Chehem F, de Mornac D, Feuillet F, Liozon E, Samson M, Bonnotte B, de Boysson H, Guffroy A, Balquet MH, Ledoult E, et al. Giant cell arteritis associated with scalp, tongue or lip necrosis: a French multicenter case control study. Semin Arthr Rheum. 2023;64:152348. 38091870 10.1016/j.semarthrit.2023.152348

[50] Régis C, Abikhzer G, Harel F, Pelletier-Galarneau M. Molecular imaging of large vessel vasculitis. J Med Imag Radiat Sci. 2023;55:S10–6. 10.1016/j.jmir.2023.11.010 38097449

[51] Watanabe R, Hashimoto M. Aging-related vascular inflammation: giant cell arteritis and neurological disorders. Front Aging Neurosci. 2022;14: 843305. 35493934 10.3389/fnagi.2022.843305 PMC9039280

[52] Zhao TV, Sato Y, Goronzy JJ, Weyand CM. T-cell aging-associated phenotypes in autoimmune disease. Front Aging. 2022;3: 867950. 35821833 10.3389/fragi.2022.867950 PMC9261367

[53] Piggott K, Biousse V, Newman NJ, Goronzy JJ, Weyand CM. Vascular damage in giant cell arteritis. Autoimmunity. 2009;42(7):596–604. 19657775 10.1080/08916930903002495 PMC4271842

[54] Greigert H, Genet C, Ramon A, Bonnotte B, Samson M. New Insights into the pathogenesis of giant cell arteritis: mechanisms involved in maintaining vascular inflammation. J Clin Med. 2022;11(10):2905. 35629030 10.3390/jcm11102905 PMC9143803

[55] Ahmed R, Gray D. Immunological memory and protective immunity: understanding their relation. Science (New York, NY). 1996;272(5258):54–60. 10.1126/science.272.5258.54 8600537

[56] Lai W-F, Wong W-T. Roles of the actin cytoskeleton in aging and age-associated diseases. Ageing Res Rev. 2020;58: 101021. 31968269 10.1016/j.arr.2020.101021

[57] van der Vorst EP, Döring Y, Weber C. MIF and CXCL12 in cardiovascular diseases: functional differences and similarities. Front Immunol. 2015;6:373. 26257740 10.3389/fimmu.2015.00373 PMC4508925

[58] Lue H, Dewor M, Leng L, Bucala R, Bernhagen J. Activation of the JNK signalling pathway by macrophage migration inhibitory factor (MIF) and dependence on CXCR4 and CD74. Cell Signal. 2011;23(1):135–44. 20807568 10.1016/j.cellsig.2010.08.013 PMC3586206

[59] Zhang Z, Zhu H, Zhao C, Liu D, Luo J, Ying Y, Zhong Y. DDIT4 promotes malignancy of head and neck squamous cell carcinoma. Mol Carcinog. 2023;62(3):332–47. 36453700 10.1002/mc.23489

[60] Song L, Chen Z, Zhang M, Zhang M, Lu X, Li C, Miao L. DDIT4 overexpression associates with poor prognosis in lung adenocarcinoma. J Cancer. 2021;12(21):6422–8. 34659532 10.7150/jca.60118 PMC8489140

[61] Lin X, Yoshikawa N, Liu W, Matsukawa T, Nakamura K, Yoshihara M, Koya Y, Sugiyama M, Tamauchi S, Ikeda Y, et al. DDIT4 facilitates lymph node metastasis via the activation of NF-κB pathway and epithelial-mesenchymal transition. Reprod Sci (Thousand Oaks, Calif). 2023;30(9):2829–41. 10.1007/s43032-023-01230-y 37016173

[62] Hou X, Yang S, Yin J. Blocking the REDD1/TXNIP axis ameliorates LPS-induced vascular endothelial cell injury through repressing oxidative stress and apoptosis. Am J Physiol Cell Physiol. 2019;316(1):C104-c110. 30485138 10.1152/ajpcell.00313.2018

[63] Lee DK, Kim JH, Kim J, Choi S, Park M, Park W, Kim S, Lee KS, Kim T, Jung J, et al. REDD-1 aggravates endotoxin-induced inflammation via atypical NF-κB activation. FASEB J Off Publ Fed Am Soc Exp Biol. 2018;32(8):4585–99. 10.1096/fj.201701436R 29547704

[64] Seoh ML, Ng CH, Yong J, Lim L, Leung T. ArhGAP15, a novel human RacGAP protein with GTPase binding property. FEBS Lett. 2003;539(1–3):131–7. 12650940 10.1016/s0014-5793(03)00213-8

[65] Fox CJ, Hammerman PS, Thompson CB. Fuel feeds function: energy metabolism and the T-cell response. Nat Rev Immunol. 2005;5(11):844–52. 16239903 10.1038/nri1710

[66] Yang C, Ko B, Hensley CT, Jiang L, Wasti AT, Kim J, Sudderth J, Calvaruso MA, Lumata L, Mitsche M, et al. Glutamine oxidation maintains the TCA cycle and cell survival during impaired mitochondrial pyruvate transport. Mol Cell. 2014;56(3):414–24. 25458842 10.1016/j.molcel.2014.09.025 PMC4268166

[67] Song M, Sandoval TA, Chae CS, Chopra S, Tan C, Rutkowski MR, Raundhal M, Chaurio RA, Payne KK, Konrad C, et al. IRE1α-XBP1 controls T cell function in ovarian cancer by regulating mitochondrial activity. Nature. 2018;562(7727):423–8. 30305738 10.1038/s41586-018-0597-x PMC6237282

[68] Carr EL, Kelman A, Wu GS, Gopaul R, Senkevitch E, Aghvanyan A, Turay AM, Frauwirth KA. Glutamine uptake and metabolism are coordinately regulated by ERK/MAPK during T lymphocyte activation. J Immunol. 2010;185(2):1037–44. 20554958 10.4049/jimmunol.0903586 PMC2897897

[69] Fang X, Xie M, Liu X, He Y. REDD1 gene knockout alleviates vascular smooth muscle cell remodeling in pulmonary hypertension. Am J Trans Res. 2022;14(3):1578–91. PMC8991113 35422917

[70] Wenes M, Shang M, Di Matteo M, Goveia J, Martín-Pérez R, Serneels J, Prenen H, Ghesquière B, Carmeliet P, Mazzone M. Macrophage metabolism controls tumor blood vessel morphogenesis and metastasis. Cell Metab. 2016;24(5):701–15. 27773694 10.1016/j.cmet.2016.09.008

[71] Stevens SA, Gonzalez Aguiar MK, Toro AL, Yerlikaya EI, Sunilkumar S, VanCleave AM, Pfleger J, Bradley EA, Kimball SR, Dennis MD. PERK/ATF4-dependent expression of the stress response protein REDD1 promotes proinflammatory cytokine expression in the heart of obese mice. Am J Physiol Endocrinol Metab. 2023;324(1):E62-e72. 36383638 10.1152/ajpendo.00238.2022 PMC9870577

