# Supplementary material for: Causal genes identification of giant cell arteritis in CD4+ Memory t cells: an integration of multi-omics and expression quantitative trait locus analysis
Source: Inflamm Res. 2025 Jan 7;74(1):3. doi: 10.1007/s00011-024-01965-7 (PMC11703992; doi:10.1007/s00011-024-01965-7)
Supplement: Supplementary file 2 — Supplementary Material 2 [file 11_2024_1965_MOESM2_ESM.docx]

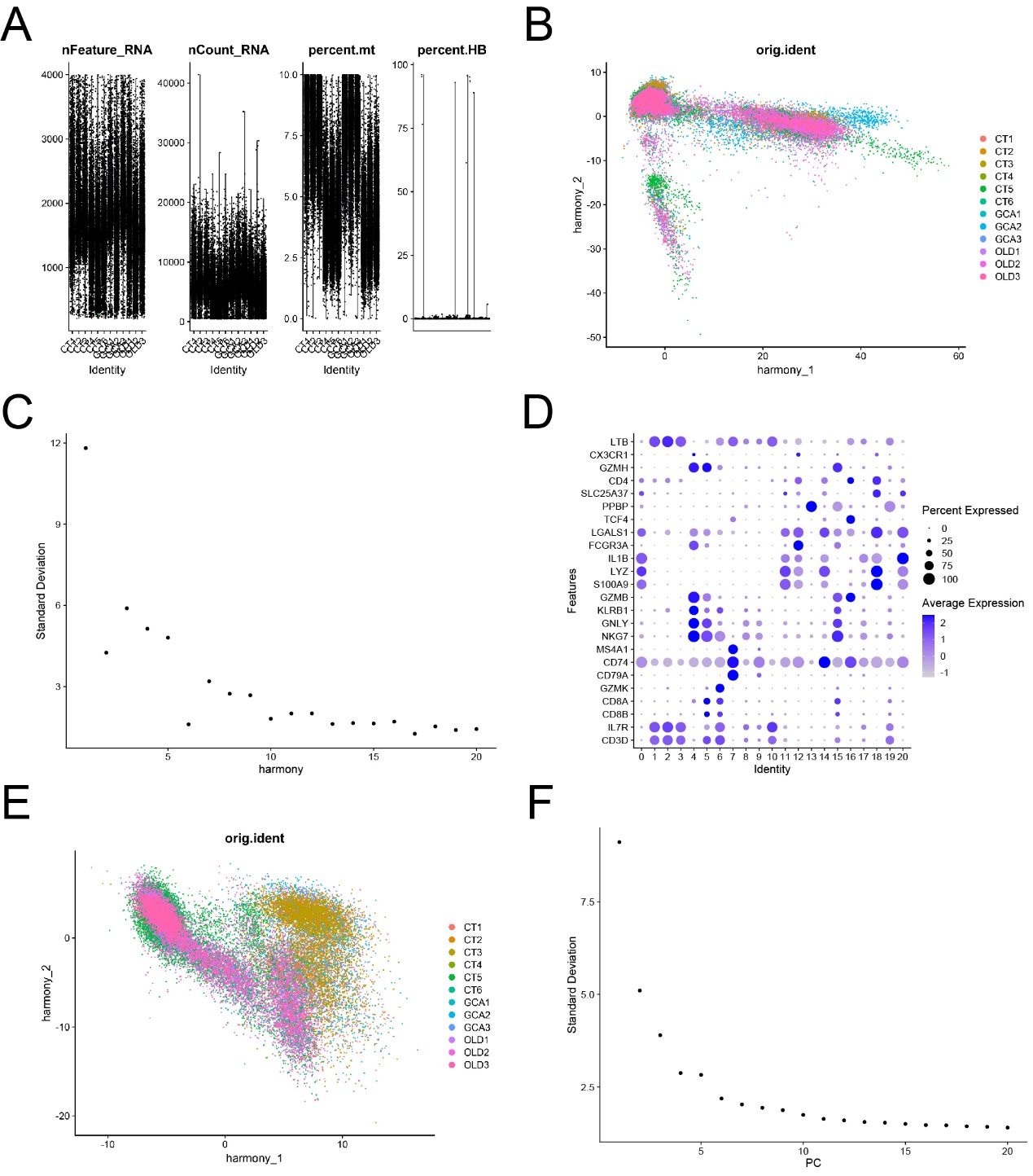


**Fig. S1** The pre-processing of scRNA data. (A) Quality control of scRNA data for control, old and GCA samples. (B) Scatter plot showing the scores of individual cells (points) along the top two harmony dimensions. (C) Standard deviation (y-axis) accounts for top 20 PCs (x-axis) to identify the number of significant PCs based on the presence of an “elbow”. Approximately 10 PCS are chosen for the analysis. (D) Dotplot showing the average expression level of canonical marker genes of each cluster in integrated scRNA samples. (E) Scatter plot showing the scores of each extracted T cells (points) along the top two harmony dimensions. (F) Standard deviation (y-axis) accounts for top 20 PCs (x-axis) of T cells to identify the number of significant PCs based on the presence of an “elbow”. Approximately 10 PCS are chosen.


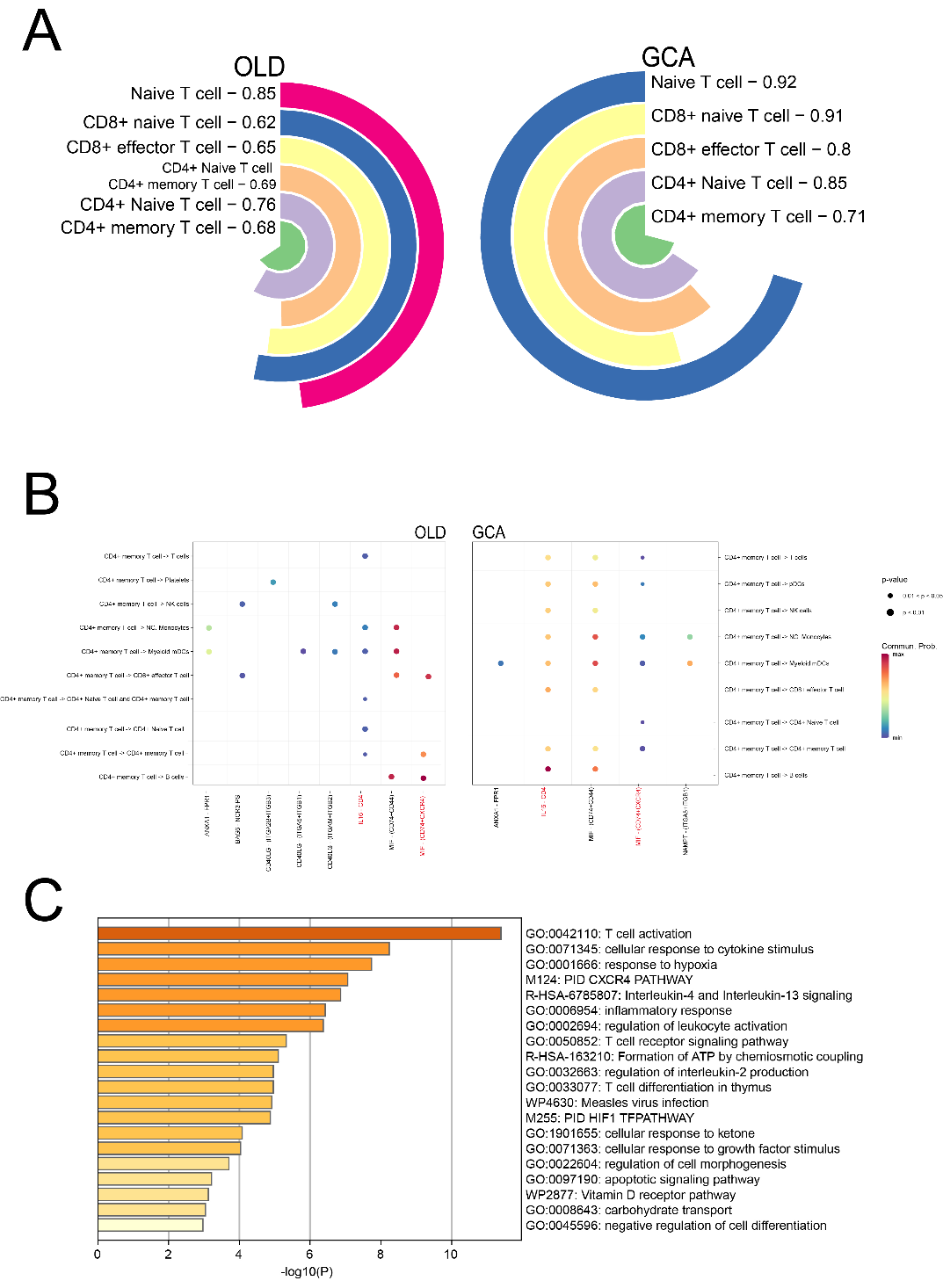


**Fig. S2** CD4+ Memory T cells in GCA and old samples. (A) Cell perturbation of different cell clusters to aging and GCA, evaluated by Augur method. (B) The major communication pathways of CD4+ Memory T cells in old samples and GCA samples. Significant pathways were marked red. (C) Functional enrichment analysis of DEGs in CD4+ Memory T cells in GCA.


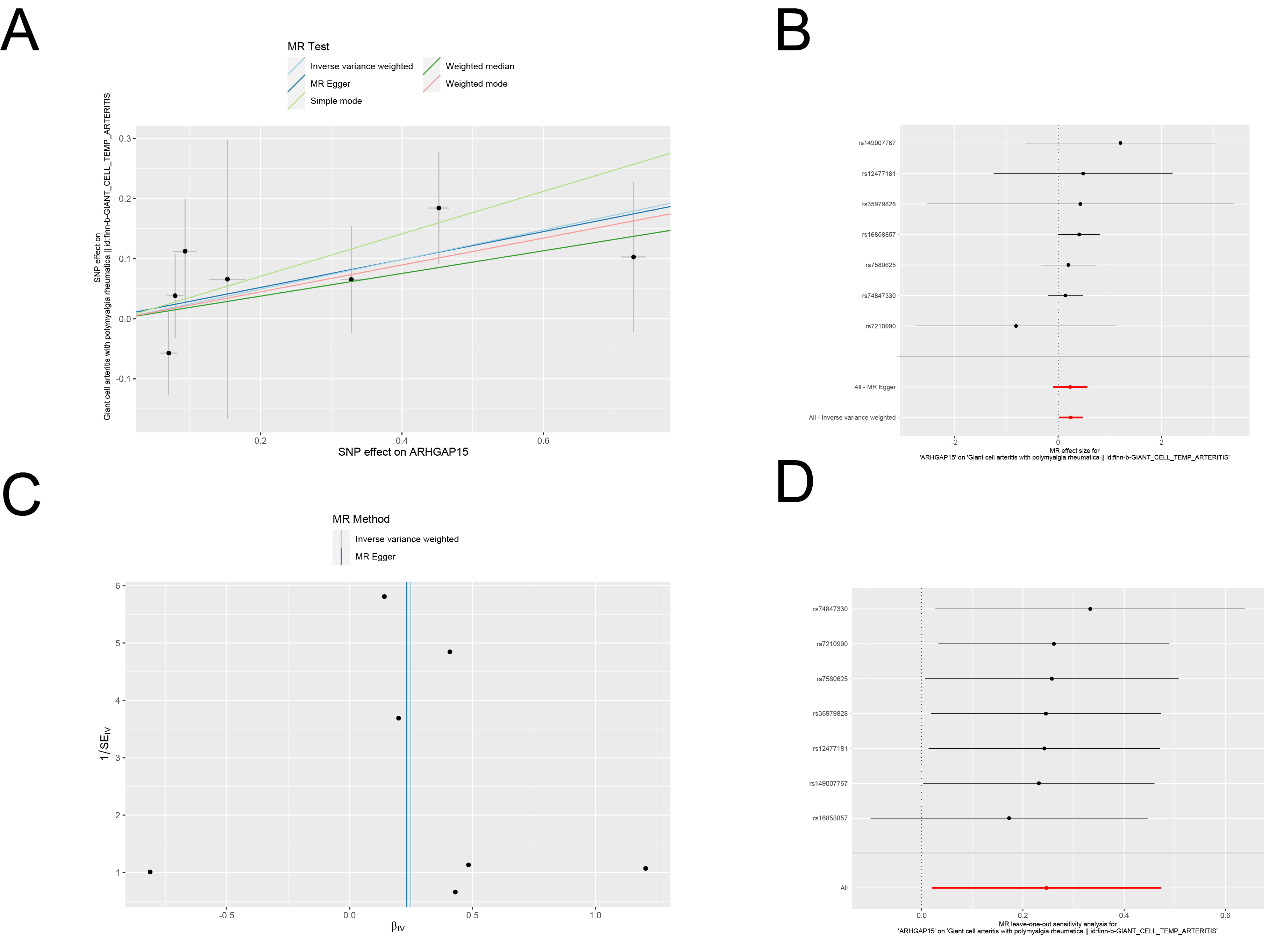


**Fig. S3** The MR analysis process of ARHGAP15 in dataset finn-b-GIANT_CELL_TEMP_ARTERITIS. (A) Scatter plot showing the causal effect of ARHGAP15 on the risk of GCA in validation dataset. (B) Forest plot showing the causal effect of each SNP on the risk of GCA. (C) Funnel plots visualized overall heterogeneity of MR estimates for the effect of ARHGAP15 on GCA. (D) Leave-one-out plot indicated causal effect of ARHGAP15 on GCA risk when leaving one SNP out.


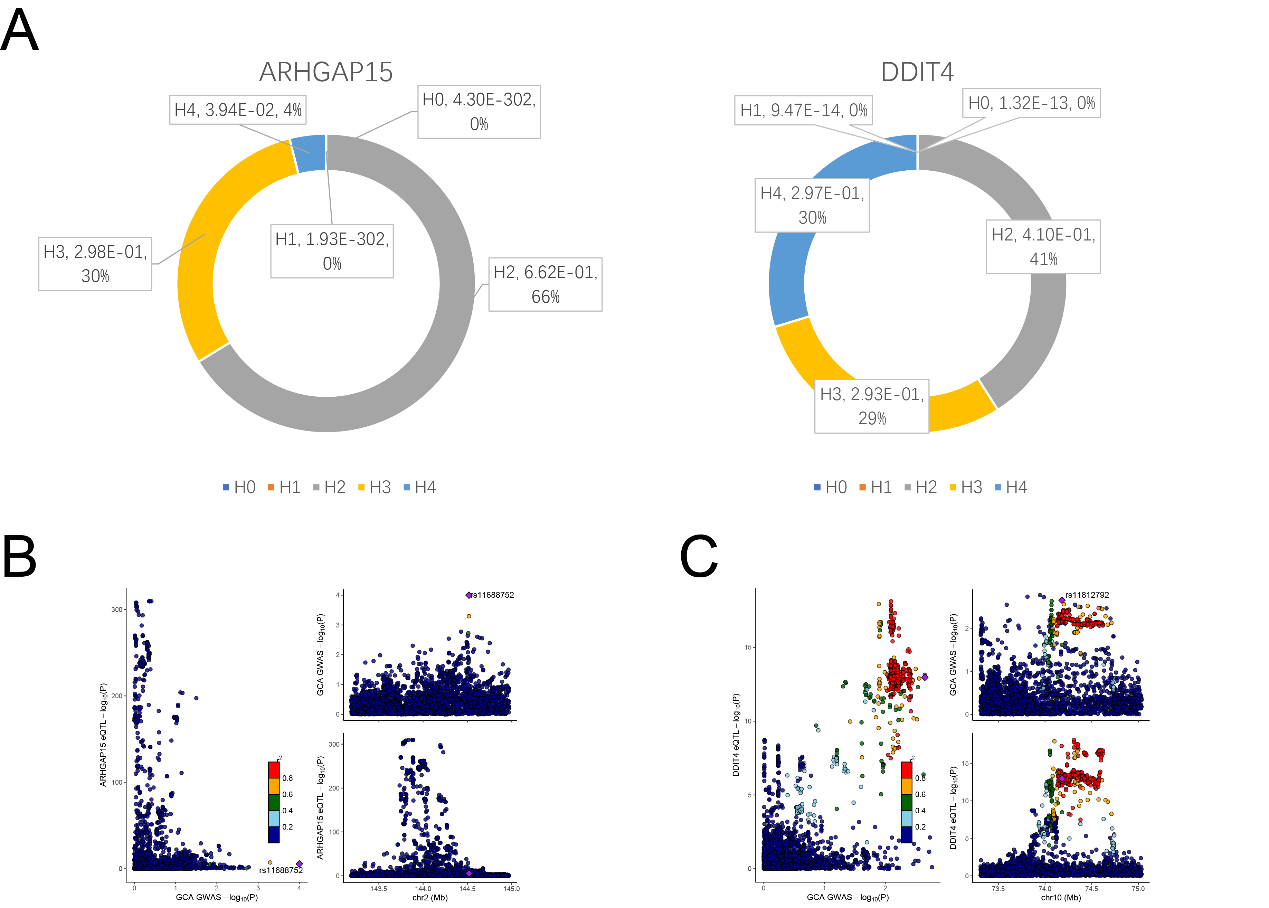


**Fig. S4** The colocalization analysis of eQTLs for DDIT4 and ARHGAP15 with GCA-associated SNPs. (A) The posterior probabilities of ARHGAP15 and DDIT4 for five hypotheses, which are H0, H1, H2, H3, H4. (B, C) The locus comparisons between ARHGAP15 or DDIT4 eQTLs and GCA GWAS by colocalization analysis. The r^2^ value indicates the linkage disequilibrium (LD) between the variants and the top SNPs.


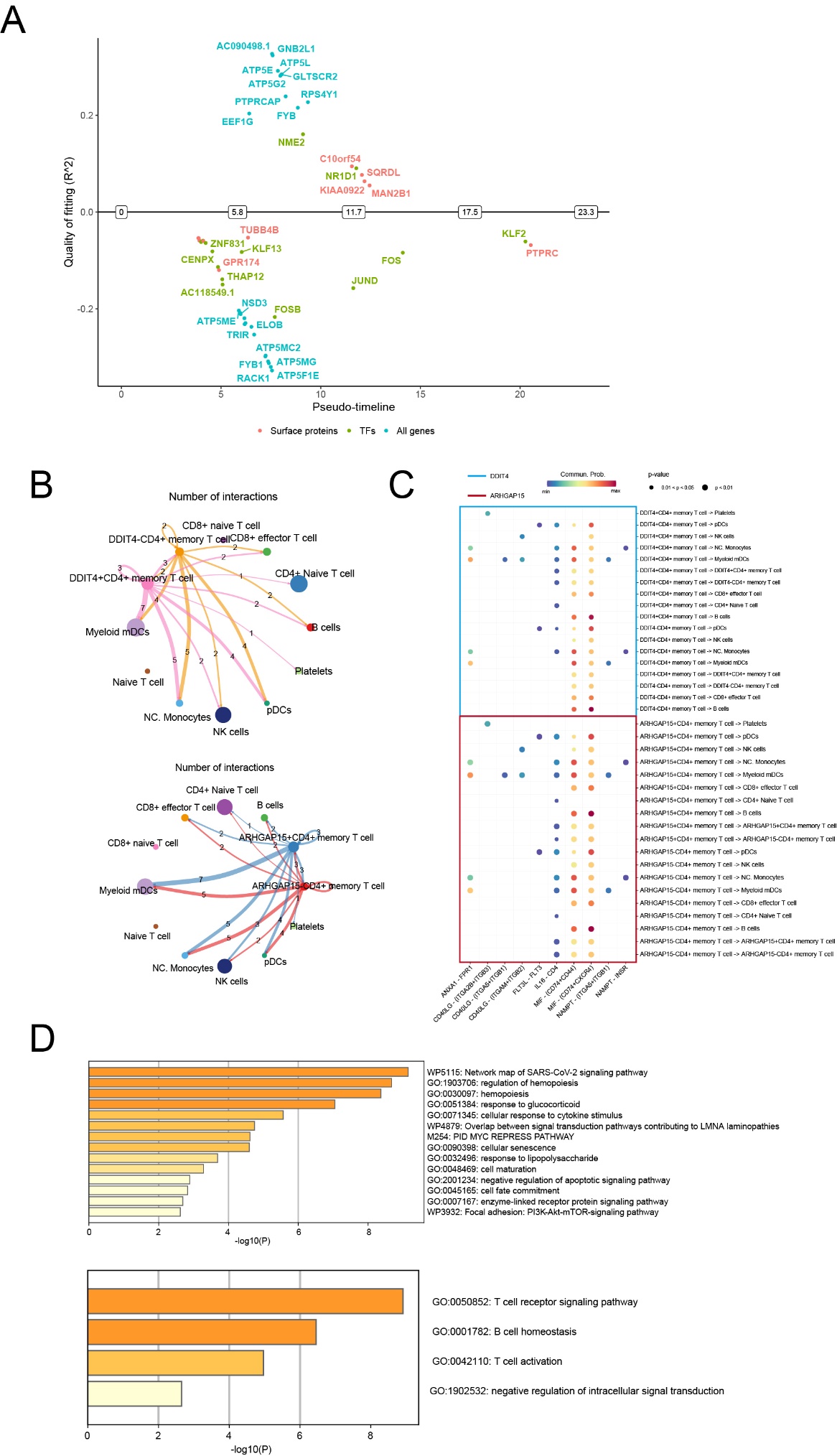


**Fig. S5** DDIT4 and ARHGAP15 in CD4+ Memory T cells. (A) GeneSwitches analysis of scRNA-seq data. Visualization of the order of top fitting switching genes from various sets of known proteins along the pseudotime. The absolute value of the y-axis is the quality of fitting defined by McFadden's Pseudo R2, and the positive and negative signs indicate up- and down-regulation respectively. (B, C) The differences of cell-cell communication and major pathways between DDIT4+ and DDIT4- CD4+ Memory T cells, as well as ARHGAP15+ and ARHGAP15- CD4+ Memory T cells. In Fig. S5C, blue represents DDIT4+ and DDIT4- groups, and red represents ARHGAP15+ and ARHGAP15- groups. (D) The functional enrichment of differentially expressed genes between DDIT4+/- groups and ARHGAP15+/- groups.


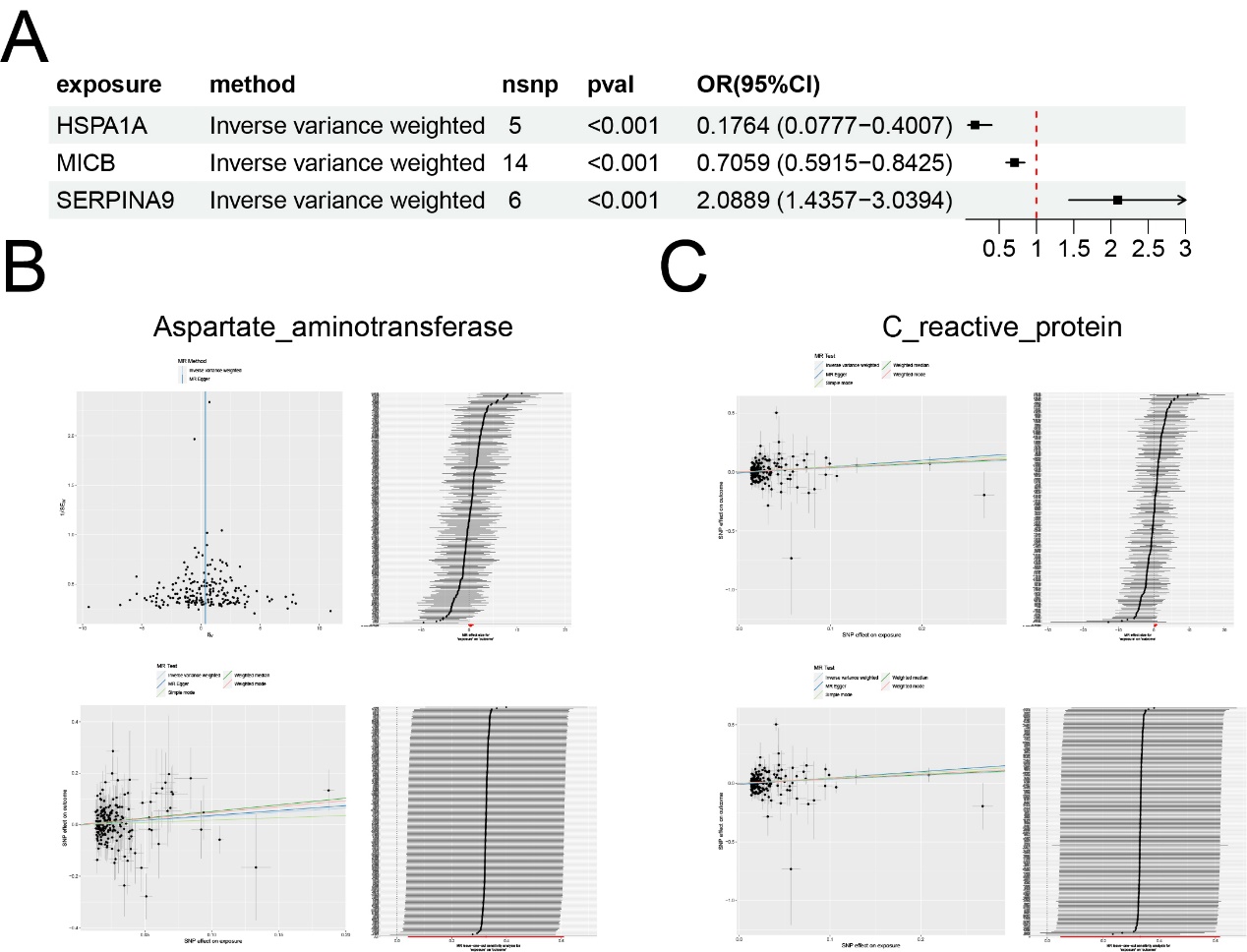


**Fig. S6** Causal proteins and biomarkers related to GCA occurrence. (A) Plasma proteins that have causal associations with GCA after Bonferroni adjustment, p < 0.001. (B, C) MR analysis of blood-urine biomarkers aspartate aminotransferase and C reactive protein with GCA risk.


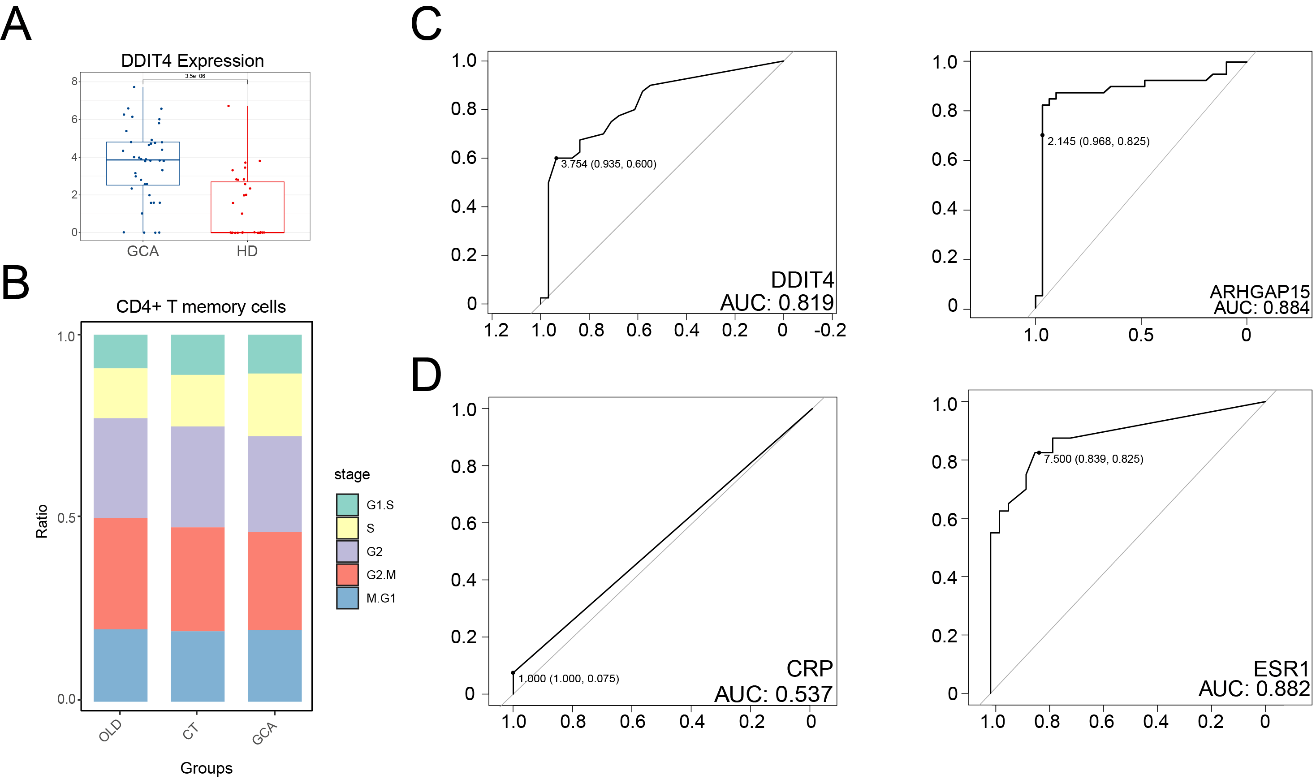


**Fig. S7** Bulk-seq validation, and cell cycle analysis of CD4+ Memory T cells in GCA. (A) The expression of DDIT4 in GCA groups and control groups. (B) The cell cycle analysis of CD4+ Memory T cells in GCA, CT and old samples. (C, D) The prediction ability of diagnosis via DDIT4, ARHGAP15 and typical marker CRP, ESR1 respectively measured by ROC curves.


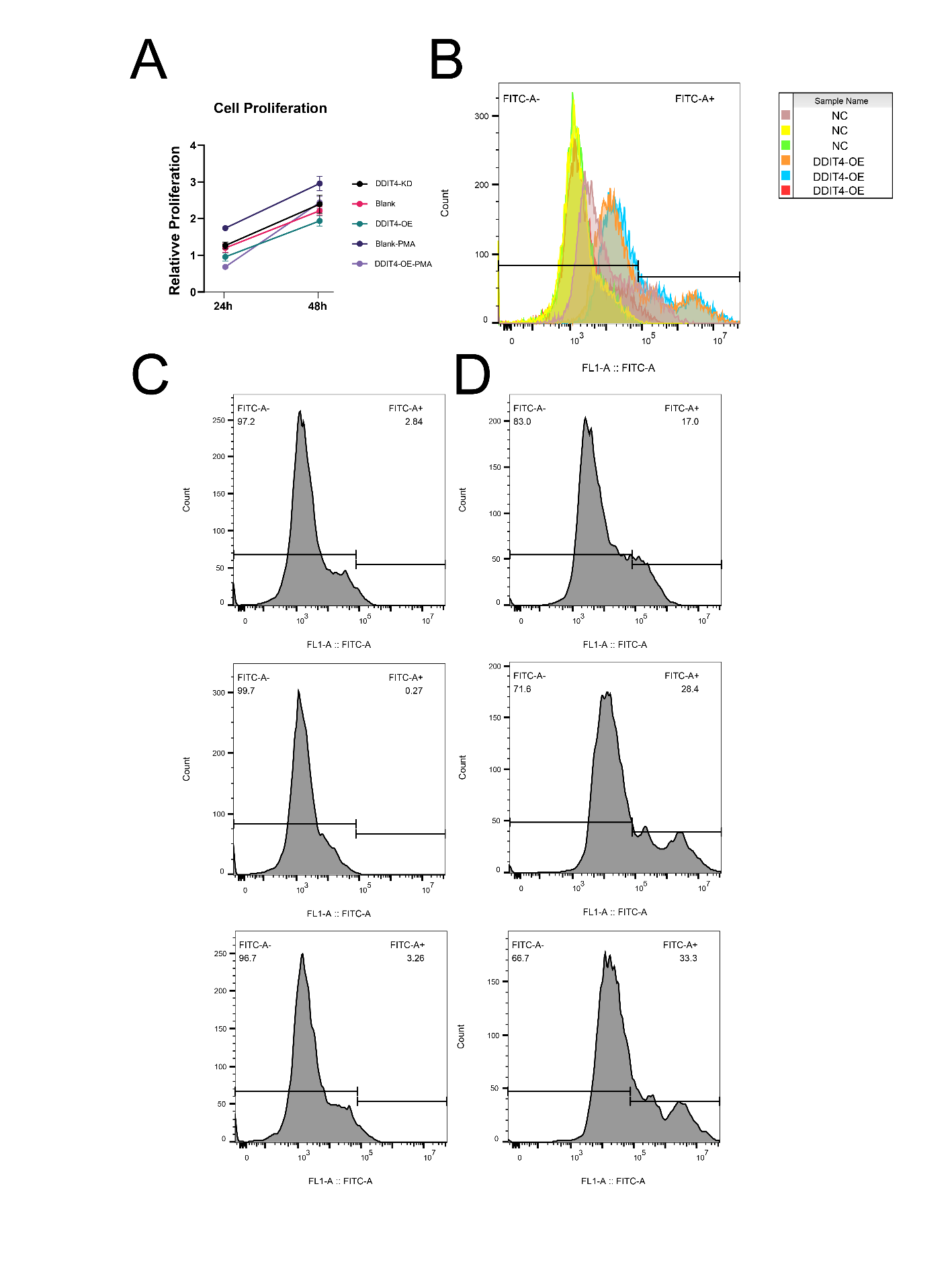


**Fig. S8** The objective of this study is to examine the cell proliferative capacity and expression of CD45 differentiation markers in Jurkat cells with DDIT4 overexpression. (A) Cell proliferation activity assays for different groups of Jurkat cells. (B) The objective of this study is to detect CD45 differentiation markers in Jurkat cells of DDIT4-OE using flow cytometry. (C) The objective of this study is to detect CD45 differentiation markers in normal Jurkat cells using flow cytometry. (D) The objective of this study was to detect CD45 differentiation markers in DDIT4-OE Jurkat cells using flow cytometry.


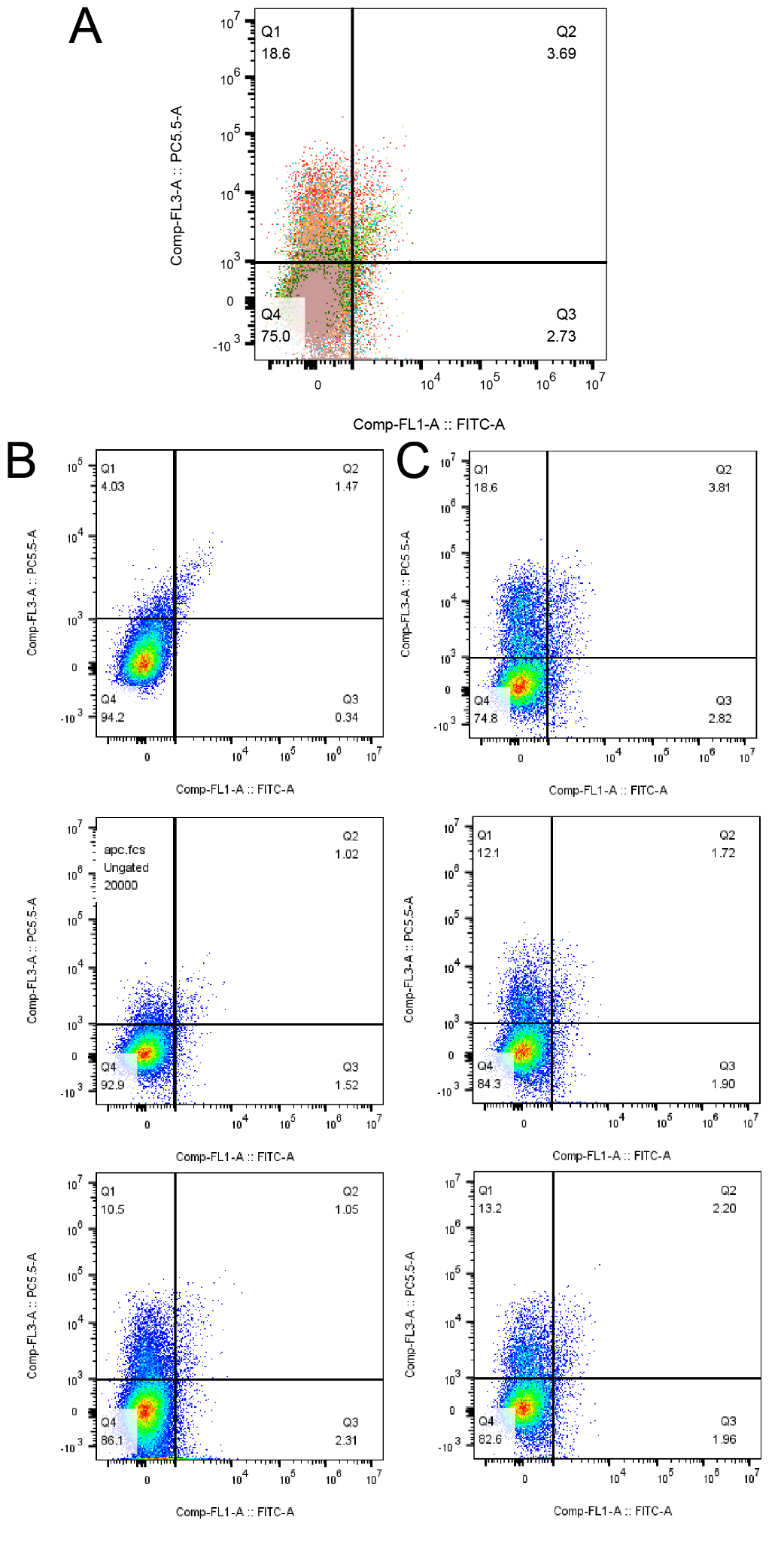


**Fig. S9** Apoptosis of Jurkat cells after 48 hours of PMA treatment. (A) Overlapping images of all samples. (B) Apoptosis in DDIT4-OE-PMA Jurkat cells. (C) Apoptosis in blank-PMA Jurkat cells of DDIT4-OE.


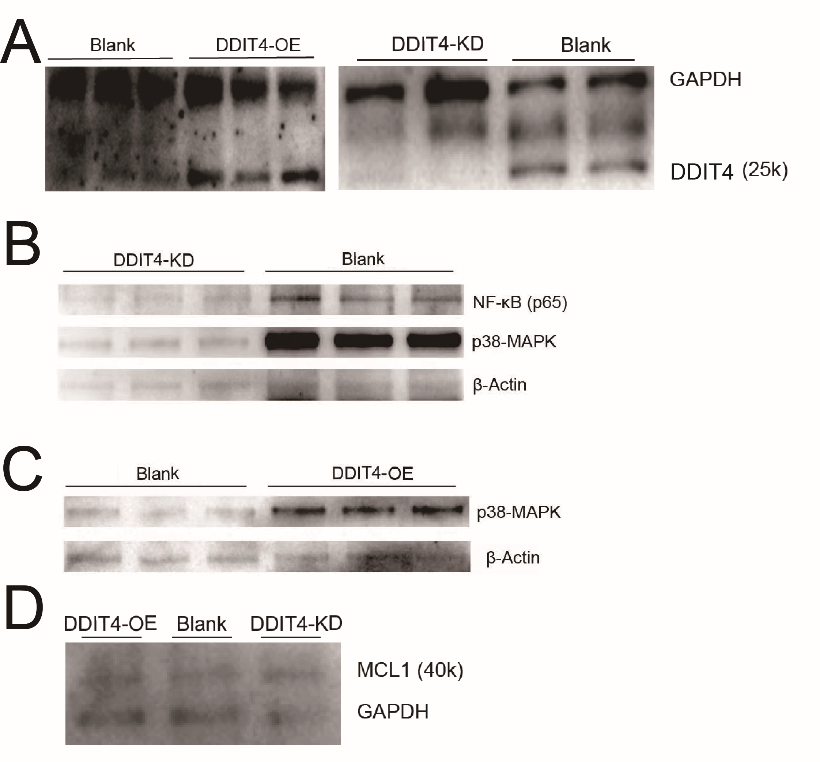


**Fig. S10** Result of WB. (A) Expression of DDIT4 in Jurkat cells with DDIT4-OE/KD. (B) NF-κB, p38-MAPK expression in DDIT4-KD and blank Jurkat cells. (C) p38-MAPK expression in DDIT4-OE and blank Jurkat cells. (D) MCL-1 expression in DDIT4-OE, DDIT4-KD and blank Jurkat cells.

**pIRES2-EGFP-DDIT4**


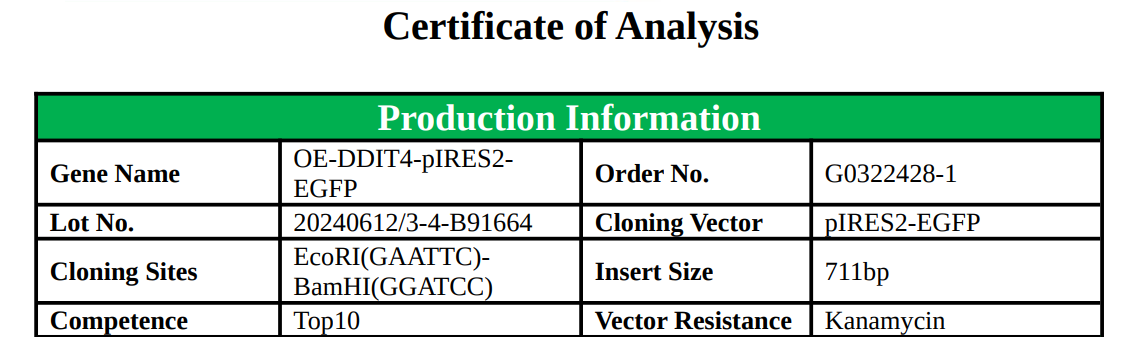


GAATTCATGCCTAGCCTTTGGGACCGCTTCTCGTCGTCGTCCACCTCCTCTTCGCCCTCGTCCTTGCCCCGAACTCCCACCCCAGATCGGCCGCCGCGCTCAGCCTGGGGGTCGGCGACCCGGGAGGAGGGGTTTGACCGCTCCACGAGCCTGGAGAGCTCGGACTGCGAGTCCCTGGACAGCAGCAACAGTGGCTTCGGGCCGGAGGAAGACACGGCTTACCTGGATGGGGTGTCGTTGCCCGACTTCGAGCTGCTCAGTGACCCTGAGGATGAACACTTGTGTGCCAACCTGATGCAGCTGCTGCAGGAGAGCCTGGCCCAGGCGCGGCTGGGCTCTCGACGCCCTGCGCGCCTGCTGATGCCTAGCCAGTTGGTAAGCCAGGTGGGCAAAGAACTACTGCGCCTGGCCTACAGCGAGCCGTGCGGCCTGCGGGGGGCGCTGCTGGACGTCTGCGTGGAGCAGGGCAAGAGCTGCCACAGCGTGGGCCAGCTGGCACTCGACCCCAGCCTGGTGCCCACCTTCCAGCTGACCCTCGTGCTGCGCCTGGACTCACGACTCTGGCCCAAGATCCAGGGGCTGTTTAGCTCCGCCAACTCTCCCTTCCTCCCTGGCTTCAGCCAGTCCCTGACGCTGAGCACTGGCTTCCGAGTCATCAAGAAGAAGCTGTACAGCTCGGAACAGCTGCTCATTGAGGAGTGTTGAGGATCC

**pKDL-DDIT4**


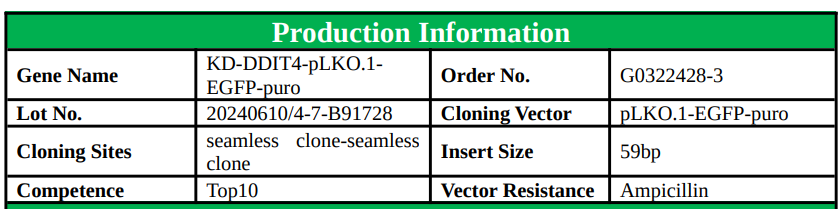


CCGGTGTGTAGCATGTACCTTATTACTCGAGTAATAAGGTACATGCTACACATTTTTTG
